# Survival Rates of Short Dental Implants (≤6 mm) Used as an Alternative to Longer (>6 mm) Implants for the Rehabilitation of Posterior Partial Edentulism: A Systematic Review of RCTs

**DOI:** 10.3390/dj12060185

**Published:** 2024-06-17

**Authors:** Rodopi Emfietzoglou, Xanthippi Dereka

**Affiliations:** Department of Periodontology, School of Dentistry, National and Kapodistrian University of Athens, 11527 Athens, Greece; r.emfietzoglou@gmail.com

**Keywords:** dental implantation, alveolar bone loss, alveolar ridge augmentation, survival rate, postoperative complications, peri-implantitis

## Abstract

Short dental implants have been proposed as an alternative treatment option to bone regeneration procedures for the rehabilitation of resorbed alveolar ridges. The aim of this paper was to systematically review randomized controlled trials (RCTs) comparing short implants (≤6 mm) and longer implants (>6 mm) in atrophic alveolar ridges in terms of implant survival rates, peri-implant marginal bone loss (MBL), prevalence of peri-implantitis and technical complications. A thorough electronic search was performed until September 2023. RCTs with follow-up of at least 1-year post-loading comparing short implants with rough surfaces to longer implants in the posterior jaws of systemically and periodontally healthy, partially edentulous adults were considered. Studies with incomplete information on the number of patients, follow-up or definition of “short implants” were excluded. The revised Cochrane risk-of-bias tool for randomized trials was used for Risk of bias assessment. Fixed-effects meta-analysis of the selected studies was applied to compare the outcome variables. Random-effect meta-analysis was performed, on the basis of within-study comparisons. In total, 16 articles were selected for meta-analysis and incorporated 408 short implants and 475 longer implants inserted in 317 and 388 patients, respectively. The survival rates of longer implants in pristine or augmented bone were significantly increased compared to short implants (95%CI: 2–5%, *p* < 0.001). Standard-length implants displayed increased, although non-statistically significant MBL (95%CI: −0.17–0.04, *p* > 0.05), and prevalence of peri-implantitis (95%CI: 0–5%, *p* > 0.05). No statistically significant differences were observed between short and long implants in terms of technical complications (implant-level 95%CI: −4–6%, *p* > 0.05). Short implants represent a promising alternative treatment option for the rehabilitation of posterior jaws to avoid additional bone augmentation procedures. Nonetheless, they should be selected cautiously due to a potentially limited survival rate compared to longer implants. A major limitation of this study is the variability in the included studies regarding sample size, patient profile, type of bone, loading protocol, definition of peri-implantitis, among others. This study received no external funding. The study protocol was registered in PROSPERO (CRD42023485514).

## 1. Introduction

Dental implants represent a clinically effective treatment option for the rehabilitation of full or partial edentulism. Periodontal diseases, trauma, sinus expansion and inferior alveolar nerve may limit the bone height of maxilla and mandible. Thus, the alveolar ridge may be unfavorable for the insertion of implants of standard length. Several advanced regenerative procedures, including sinus lift elevation, guided bone regeneration, distraction osteogenesis, onlay bone grafting as well as displacement of the inferior alveolar nerve may be performed in patients with reduced alveolar bone height [1]. An interesting technique that has been recently suggested concerning implant placement in the posterior atrophic maxilla, involves drilling of implant sites at various depths to enable apical bone displacement, crestal sinus membrane elevation, and the placement of a spiral-shaped implant longer than pre-surgical radiographic measurements [2]. It has been reported that implant placement in the atrophic posterior maxilla along with sinus elevation, without using bone grafts may lead to substantial bone formation around the implants at the sinus floor, resulting in successful restorations and negating the need for bone grafts [3].

These surgical techniques, as well known, are linked to increased risk of postoperative morbidity high economic cost and prolonged treatment duration and complications [4,5]. For instance, complications that should be taken into account when sinus augmentation is needed for implant placement in the posterior maxilla include tearing of the Schneiderian membrane, antral or nasal penetration, fenestration, dehiscence or perforation of the alveolar bone, bleeding, pain, edema, hemosinus, sinusitis, graft or implant migration in the sinus, and oroantral fistula [2,3]. On the other hand, some surgical procedures might not be allowed in systemically compromised patients [6]. The use of dental implants with reduced length (short implants) has been suggested as an alternative for the treatment of atrophic alveolar ridge.

Several definitions of short implants have been suggested in different studies. Some authors consider as short, implants with length less than 7 mm [7], 8 mm [1], 10 mm [8], or even 11 mm [9]. Within this review, the term “short implants” refers to implants with intrabony length ≤ 6 mm, according to the 6th ITI Consensus Conference [10].

The survival of short implants is a topic with conflicting results among the studies. Papaspyridakos et al. indicated that mean survival rate was 96% (range: 86.7–100%) for short implants (≤6 mm) in posterior jaws, and 98% (range 95–100%) for longer implants after 1 to 5 years in function [11]. According to the 6th ITI Consensus Conference, the mean survival rates for short and longer implants placed in posterior jaws were 96% (range: 86.7–100%) and 98% (range 95–100%), respectively, after 1–5 years in function [10].

According to the 6th ITI Consensus Conference, short and standard-length implants in posterior jaws present similar radiographic interproximal bone level alterations after 1–5 years, which ranged from +0.06 to −1.22 mm and from +0.02 to −1.54 mm for short and longer implants, respectively [10]. However, other recent systematic reviews have observed significantly higher marginal bone loss (MBL) around longer implants compared to short implants placed in pristine or augmented bone [4,12,13].

Short dental implants are associated with a shorter surgical and healing phase, reduced morbidity, and treatment cost [6,14], but present a higher risk of occlusal overload compared to standard implants [10].

Despite the large number of available systematic reviews comparing the outcomes of short and longer implants inserted in different jaw areas and clinical scenarios (e.g., immediate or delayed loading, pristine or augmented bone, etc.), scarce studies examine implant surface characteristics. Moderately rough and rough implant surfaces present an increased surface area for fibrin attachment, which promotes the migration of bone progenitor cells and, therefore, bone apposition adjacent to the implant. This results in increased levels of bone-to-implant-contact and enhanced osseointegration [15,16,17].

Hence, the aim of this paper was to systematically review randomized controlled trials (RCTs) that compare short implants (≤6 mm) with rough surface and longer implants (>6 mm) in atrophic alveolar ridge in terms of implant survival rates, peri-implant marginal bone loss, prevalence of peri-implantitis, and technical complications.

## 2. Materials and Methods

This review was conducted according to the Preferred Reporting Items for Systematic Reviews and Meta-Analyses (PRISMA) statement [18]. The study protocol was registered in PROSPERO (CRD42023485514) and is in line with the Cochrane handbook [19]. No significant amendments have been made to the protocol.

### 2.1. Focused Question

The focused question for this review based on the PICOS [20] was, “Is there a difference in the survival rate of short implants (≤6 mm) with rough surfaces as compared with implants > 6 mm long after 12 months of loading?”.

### 2.2. PICO

- Population: systemically and periodontally healthy, partially edentulous adult subjects (≥18 years old) with implant restorations in the posterior mandible or maxilla.
- Intervention (test group): studies evaluating the use of implants with rough surfaces and ≤6 mm in length.
- Comparison (control group): patients receiving dental implants > 6 mm in length and rough surfaces.
- Outcome: primary outcome: implant survival rates; secondary outcomes: radiographic marginal bone loss, prevalence of peri-implantitis, and prosthetic/technical complications.

### 2.3. Inclusion Criteria

- RCTs reporting on short and standard-length implant placement in the posterior mandible or maxilla of partially edentulous, systemically and periodontally healthy adult (≥18 years old) subjects, with a follow-up of at least 1-year post-loading.
- Studies including a minimum of 10 patients per arm and written in the English language.
- Studies comparing the outcomes of short (≤6 mm) rough-surfaced implants to standard-length implants.

### 2.4. Exclusion Criteria

- In vitro and pre-clinical studies, case reports, case series, prospective, cohort, or retrospective studies.
- Studies with follow-up < 12 months post-loading.
- Studies with incomplete information on the number of patients, follow-up, site of implant placement, implant surface characteristics, or definition of “short implants”.
- Studies including implants with length > 6 mm in the short implants group, or using non rough-surfaced implants.
- Studies not reporting on implant survival rates in short and standard-length implant groups.

### 2.5. Search Methodology

A thorough electronic search was performed in MEDLINE (PubMed) and EMBASE (OVID) in September 2023 that combined the use of MeSH terms and free text combined with the Boolean operators “AND” and “OR”. The search strategy is described in detail in Appendix A. In brief, the terms used in the electronic search were “dental implant”, “oral implant”, “osseointegrated implant”, and “short implant”. In MEDLINE, we additionally used the Boolean Operator “NOT” combined with the search terms “review” and “animal”, to exclude reviews and animal studies, respectively. The filter applied in MEDLINE was English and the results were sorted by most recent. The search terms used wereof the Journal of Clinical Periodontology, Journal of Dental Research, Journal of Periodontology, Journal of Periodontal Research, and Journal of Investigative and Clinical Dentistry were hand-searched. The references of review articles on this topic and all included studies for data extraction were screened for additional eligible papers. The search was performed independently by the two authors (R.E., X.D.).

### 2.6. Study Selection

A two-stage screening was performed in duplicate and independently by the two authors (R.E., X.D.). Studies were evaluated based on their titles and abstracts first, and studies that met the inclusion criteria were then screened for full-text evaluation. The researchers (R.E., X.D.) were blinded to each other’s decisions. Any disagreements during the above stages of the search were settled by discussion. Agreement at each of the two-stage screening processes was calculated using Kappa statistics. Decisions were recorded in an Excel spreadsheet.

### 2.7. Data Extraction

The reviewers (R.E., X.D.), who were the authors of this paper, independently extracted and recorded study data. If any information was missing or unclear, the authors were contacted by email to provide clarification or missing information. If data were missing or incomplete and further clarification from the study authors could not be obtained, such data were excluded from the analyses.

Τhe data recorded from each publication included the authors’ names, publication year, study design, follow-up periods, source of recruitment, funding status, operator, population characteristics, treatment, implant characteristics, and surgical and prosthetic parameters. Data on the primary and secondary outcomes were recorded.

### 2.8. Quality Assessment

Quality assessment and risk of bias of included papers were performed by the two reviewers (R.E., X.D.) independently according to the recommendations of the Cochrane Handbook for Systematic Reviews. The revised Cochrane risk-of-bias tool for randomized trials (RoB2) for RCTs was used [21]. Each study was evaluated to be at a low, moderate, or high risk of bias based on five domains: bias arising from the randomization process; bias due to deviations from intended interventions; bias due to missing outcome data; bias in measurement of the outcome; bias in selection of the reported result. Any disagreements between the reviewers (R.E., X.D.) were resolved by discussion. After the domain-level judgement, the overall risk of bias of each of the included studies was classified as follows: low risk of bias, some concerns, or high risk of bias. Primary and secondary outcomes were assessed for risk of bias. Articles reporting results from the same study were grouped together during quality assessment.

### 2.9. Statistical Analysis

Fixed-effects and random-effect meta-analyses of the selected studies were applied. Concerning the meta-analysis of survival rates (%) and peri-implantitis rates (%), each study data point consisted of the difference in rates between short and long groups, the number of cases, and the total population. Studies with a difference of 0 in survival and peri-implantitis rates were automatically excluded from the meta-analysis. Regarding the meta-analysis of MBL (mm), each RCT consisted of the difference in mean values between short and long groups and the standard deviation (SD), and the number of patients in each group. Early or late implant failure meta-analysis was related to risk difference. Sub-group meta-analyses were performed according to (a) follow-up period (1 year, 3 years, 5+ years); (b) baseline for MBL measurement (from loading, and from implant placement); and (c) augmented bone or pristine bone. Meta-analysis was applied to a sub-sample of the 16 studies included in the present analysis, based on surgical parameters (N = 10 studies) and implant location (maxilla) (N = 5 studies). Regarding the meta-analysis of risk difference due to technical complications, each study data point consisted of the number of patients or implants that had complications in the short and long groups. Forest plots for study-specific results were drawn. Heterogeneity was assessed with Cochran’s Q and I^2^ (I^2^ ranges between 0% and 100%, lower values represent less heterogeneity) and evaluated with the chi-square test (*p*-values < 5% were considered statistically significant). To assess the presence of publication bias, the “funnel plot” was investigated and the Egger test was carried out. All analysis was conducted using STATA version 13.0, Copyright 1985–2013 Stata Corp LP, 4905 Lake way Drive College Station, TX 77845, USA.

## 3. Results

### 3.1. Study Characteristics

**Figure 1:**
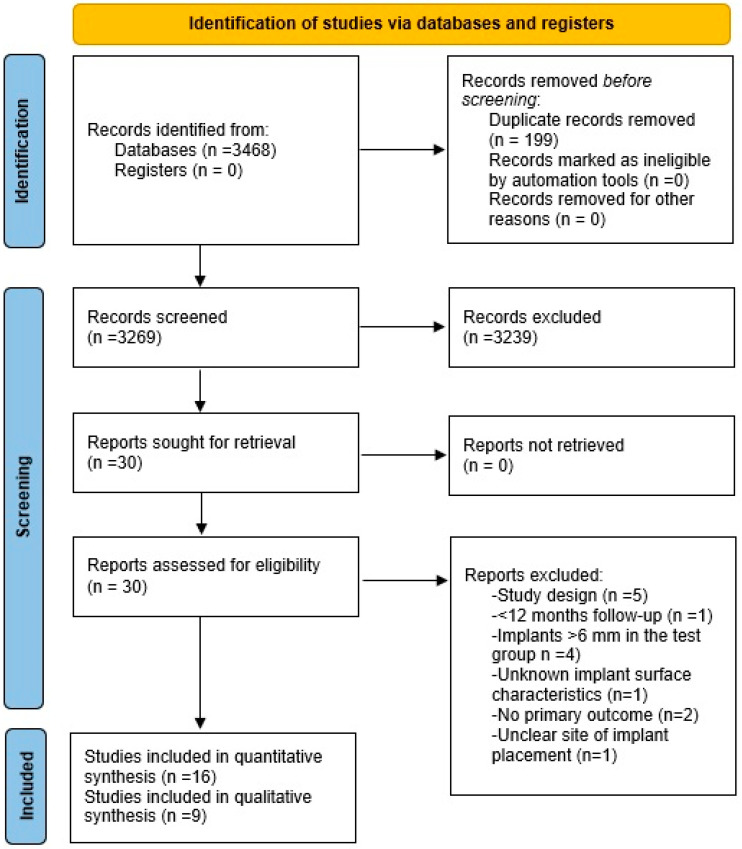
Flowchart of the study selection process (adapted from [18]).

A total of 3468 records were identified from the electronic search. The manual search did not reveal any additional records. In total, 199 records were duplicates, thus leading to 3269 unique records, which were identified and screened for titles. In total, 3049 articles were excluded, and 220 abstracts were obtained. Subsequently, 30 articles were considered eligible for full-text screening. In total, 16 articles eventually met the inclusion/exclusion criteria and were included in the analysis (Figure 1). The level of agreement between the reviewers at all stages of the screening process was high (K > 0.9).

### 3.2. Excluded Studies

Studies were excluded based on the title and the abstract mainly due to the following reasons: type of study (retrospective, prospective study, case series), enrolment of fully edentulous patients, assessment of outcomes other than the primary outcome of our study, short implants defined as >6 mm, resonance frequency analyses, follow-up < 12 months after loading, studies including implants > 6 mm in the short implants group, and studies comparing clinical outcomes of short implants placed in anterior vs. posterior regions and not short vs. standard length implants. After the full texts were obtained, five studies were excluded for reasons associated with the study design; in particular two prospective studies [22,23], two retrospective studies [24,25], and one study allowing flapless implant placement in the test and not in the control group were excluded [26]. Additional reasons for excluding studies were the following: four studies included implants with length > 6 mm in the test group [24,27,28,29], one study reported a follow-up period < 1 year [30], two studies assessed outcomes other than the ones this review evaluates [31,32], in one non-randomized clinical trial the site of implant placement was unclear [33], and in one other study the implant surface characteristics were unknown [34].

### 3.3. Included Studies

The main characteristics of the included studies are presented in Table 1 and Table 2. Overall, 16 studies reported the outcome of 408 short implants placed in 317 patients and 475 standard-length implants inserted in 388 patients. Briefly, in one study implants were placed only in the mandible [35] and in nine studies implants were placed only in the maxilla [36,37,38,39,40,41,42,43,44], whereas in the remaining six studies, implants were placed in both jaws [45,46,47,48,49,50]. Five studies had a one-year follow-up [35,39,40,44,50], four studies had a three-year follow-up [21,37,41,43,46], and seven studies had a follow-up of five years or more [36,38,42,45,47,48,49]. The length of short and standard-length implants ranged from 4 mm to 6 mm and 8 mm to 15 mm, respectively. In ten studies, the final prosthetic restoration consisted of single crowns [36,37,38,39,42,43,44,47,48,49], while in three studies the implants were restored with splinted crowns or fixed partial dentures [45,46,50]. In seven studies, the prostheses were cemented [32,35,36,37,38,40,41], and in six studies, they were screw-retained [39,45,46,48,49,50], while both retention techniques were used in three studies [42,43,44].

### 3.4. Quality Assessment

The methodological quality assessment of the nine original studies was undertaken by the ROB2 tool and is shown in Table 3 and Figure S1. One study was considered as having a high risk of bias due to bias in the measurement of outcome and deviations from intended interventions [38], and two studies as having a moderate risk of bias due to deviations from the intended interventions [36] and bias in the randomization process [47], while the remaining six studies were at a low risk of bias [35,39,41,42,45,48]. In particular, the domains related to deviations from intended interventions and randomization process raised some concerns in 33% and 11% of the studies, respectively.

### 3.5. Publication Bias

No publication bias was observed as indicated by the funnel plot which is symmetrical in shape (Figure S2). Also, no publication bias due to small-scale studies was identified, since the 95% confidence interval (CI) in the Egger test includes the value of zero (Figure S3). Small-scale studies do not alter the combined effect of the meta-analysis.

### 3.6. Implant Survival Rate

Overall, the survival rates ranged from 87% to 100% for short implants and from 97% to 100% for longer implants for follow-up periods from 1 to 10 years. Four studies reported no implant failures at 1 and 3 years after loading [37,39,43,44]. The results of the combined fixed-effect and random-effect meta-analyses showed a significantly increased survival rate in the long implant group (mean short: 95.125%, long: 99.37%, % difference: 3%, 95%CI 2–5%, *p* < 0.001, 95%CI: 3% to 7%, *p* < 0.001, respectively) (Tables S1 and S2, Figures S4 and S5). Significant heterogeneity was present: I^2^ = 62.81% and *p* < 0.001.

**Figure 2:**
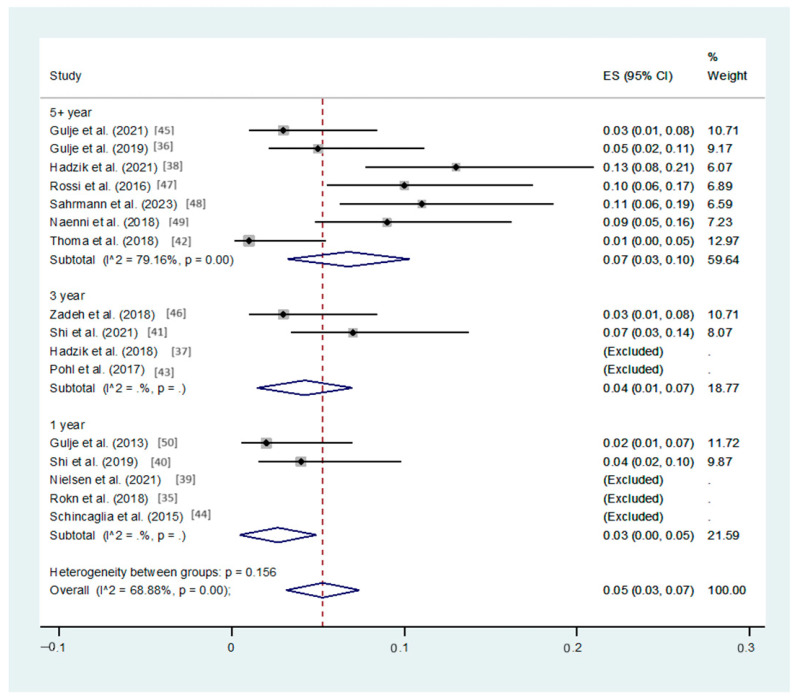
Forest plot applying random-effect meta-analysis, assessing the difference in survival rates between the short and long groups, by follow-up period (N = 16 studies) [36,37,38,39,40,41,42,43,44,45,46,47,48,49,50]. In the forestplot, the box in the middle of each horizontal line (confidence interval, CI) represents the point estimate of the effect for a single study. The size of the box is proportional to the weight of the study in relation to the pooled estimate. The diamond represents the overall effect estimate of the meta-analysis. The dashed vertical line represents the line of no effect, with the value of 0 for continuous measure such as mean difference. The placement of the center of the diamond on the x-axis represents the point estimate, and the width of the diamond represents the 95%CI around the point estimate of the pooled effect.

The combined effect analysis for a follow-up duration of 1, 3, or 5 or more years indicated a significantly increased survival rate for long implants compared to their short counterparts (95%CI: 0% to 5%, 1% to 7%, and 3% to 10%, respectively, *p*-values < 0.05). Significant heterogeneity was present in the 5+ year period studies: I^2^ = 79.16% (Figure 2).

Implant failure occurring before the abutment connection and prosthetic loading, for instance due to the inability of the host to establish osseointegration, was defined as early implant failure, while any failure after loading because of the incapacity of the host to maintain osseointegration was considered late implant failure [51,52,53].

The overall pooled estimates indicated no risk difference concerning early or late implant failure between short and long implants (95%CI: −1% to 2% and 95%CI: −1% to 2% respectively, *p*-values > 0.05). The combined-effect analysis for the 1, 3, and 5+ follow-up periods yielded no significant differences between short and long implants in terms of early and late implant failure (95%CI: −1% to 2%, −1% to 4%, −1% to 3%, respectively; *p*-values > 0.05, 95%CI: −1% to 5%, −2% to 2%, −1% to 5%, respectively; *p*-values > 0.05, I^2^ = 0% for early implant failure, I^2^ ≤ 42% late implant failure) (Figures S6 and S7).

In three studies, augmentation was performed only when necessary, in both the short and long implant groups [45,46,50]. Meta-analysis of these studies showed a significantly increased survival rate in favor of longer implants (mean short: 96.3%, long: 99%, 95%CI: 1% to 4%, *p* < 0.05) (Figure S8).

For studies assessing short implants in pristine bone and long implants in augmented bone, long implants showed a significantly increased survival rate (mean short: 96.5%, long: 99.9%, 95%CI: 2% to 9%, *p* < 0.05, I^2^ = 75.66%); however, non-significant differences were observed in the relevant studies with five or more years follow-up (95%CI: 0% to 12%, *p* > 0.05) (Figure S9).

Since only one study described short and long implant placement in pristine bone [45], sub-group analysis comparing short and long implants in pristine bone could not be performed; this also applies to secondary outcomes.

Similarly, since only one study described short and standard-length implant placement in the mandible, no sub-group analysis comparing implants in the mandible vs. maxilla could be performed. However, concerning implant placement in the maxilla, standard-length implants in vertically augmented bone presented significantly increased survival rates compared to short implants (mean short: 96.13%, long: 99.89%, 95%CI: 2% to 9%, *p* < 0.05, I^2^ = 75.66%). The combined effect for 5+ years follow-up revealed an increased survival rate for long implants (95%CI: 0% to 12%, *p* > 0.05), albeit non-statistically significant (Figure S10).

### 3.7. Marginal Bone Loss

The combined fixed-effect meta-analysis of the 16 RCTs showed a greater—but non-statistically significant—MBL in the long implant group with a mean difference of −0.09 (CI: −0.18 to 0.01, *p* = 0.082 > 0.05, I^2^ = 0%) (Figures S11 and S12, Tables S3 and S4). The mean MBL for the short implants was 0.23 mm and for long implants 0.27 mm.

**Figure 3:**
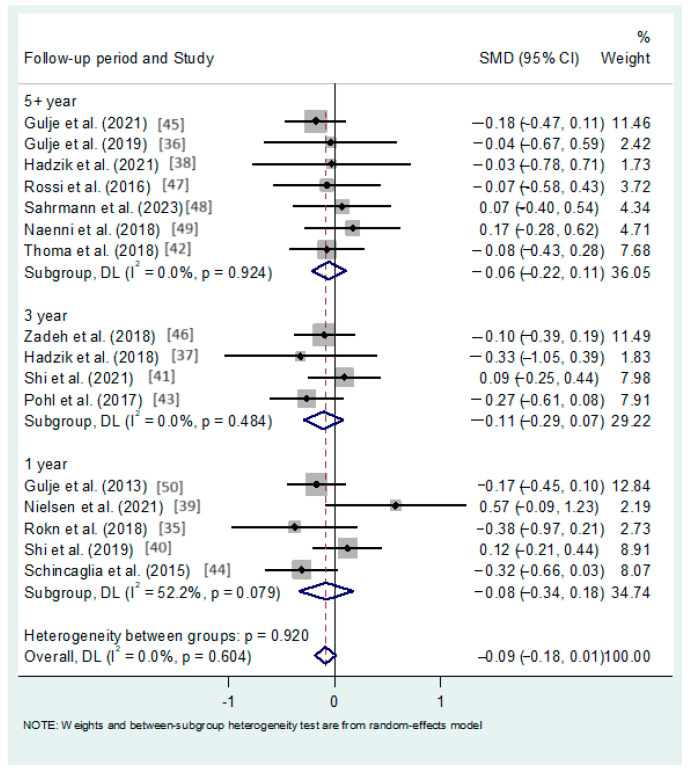
Forest plot applying random-effect meta-analysis, assessing the mean difference in MBL (mm) between the short and long groups, by follow-up period (N = 16 studies) [36,37,38,39,40,41,42,43,44,45,46,47,48,49,50]. In this forestplot, the box in the middle of each horizontal line (confidence interval, CI) represents the point estimate of the effect for a single study. The size of the box is proportional to the weight of the study in relation to the pooled estimate. The diamond represents the overall effect estimate of the meta-analysis. The dashed vertical line represents the line of no effect, with the value of 0 for continuous measure such as mean difference. The placement of the center of the diamond on the x-axis represents the point estimate, and the width of the diamond represents the 95%CI around the point estimate of the pooled effect.

The meta-analysis based on different follow-ups revealed an increased MBL for long implants and a lack of statistical significance at 1, 3, and 5 or more years post-loading, (standardized mean differences (SMDs): −0,08, −0.11 and −0.06, 95%CIs: −0.34 to 0.18; −0.29 to 0.07; −0.22 to 0.11, respectively; *p*-values > 0.05, I^2^ = 0%) (Figure 3).

Baseline marginal bone levels around implants were assessed at various intervals across different studies. Five articles conducted bone level measurements at implant placement [33,35,36,37,42], whereas the remaining eleven articles documented changes in marginal bone from prosthetic loading onwards. Changes in marginal bone levels were also compared using these two distinct baseline criteria. The combined random-effect analysis of both groups yielded increased but non-statistically significant MBL values for long implants compared to short (baseline: loading 95%CI: −0.17 to 0.04, baseline: implant placement −0.47 to 0.19; *p*-values > 0.05, I^2^ = 0%) (Figure S13).

Studies assessing short implants in pristine bone and long implants in augmented bone showed increased MBL for long implants; nevertheless, the *p*-value failed to reach statistical significance (mean short: 0.322 mm, long 0.34 mm, 95%CI: −0.23 to 0.07, *p* = 0.929 > 0.05, I^2^ = 14.5%) (Figure S14). A combined-effect analysis of these studies by follow-up period (1, 3, 5+ years) yielded similar results (95%CIs: −0.66 to 0.03; −0.38 to 0.15; −0.35 to 0.22, respectively; *p*-values > 0.05). No significant heterogeneity was present in the follow-up period of 3 and 5+ years: I^2^ = 0% to 18.2%; *p*-values > 0.05, in contrast to the studies with 1 year follow-up in which significant heterogeneity was present: I^2^ = 62.2% and *p*-value = 0.047 < 0.05.

The combined random-effect analysis of the mean difference in MBL between short implants in pristine bone and long implants in vertically augmented maxilla showed increased, although non statistically significant MBL for long implants (mean: short 0.32 mm, long: 0.36 mm, 95%CI: −0.21 to 0.10, *p* = 0.796 > 0.05, I^2^ = 15.3%) (Figure S15). The mean MBL for short implants combined-effect analysis was performed by follow-up period. Interestingly, the one-year results from three studies [37,38,42] demonstrated an increased MBL for short implants placed in the maxilla one year post-loading (95%CI: −0.38 to 0.50, *p* > 0.05, I^2^ = 69.8%). Regarding the follow-up period of 3 and 5+ years, the pooled estimate showed an increased MBL in the long implant group (95%CI: −0.38 to 0.15, and −0.35 to 0.22, respectively; *p*-values > 0.05, I^2^ = 0–18.2%).

### 3.8. Prevalence of Peri-Implantitis

**Figure 4:**
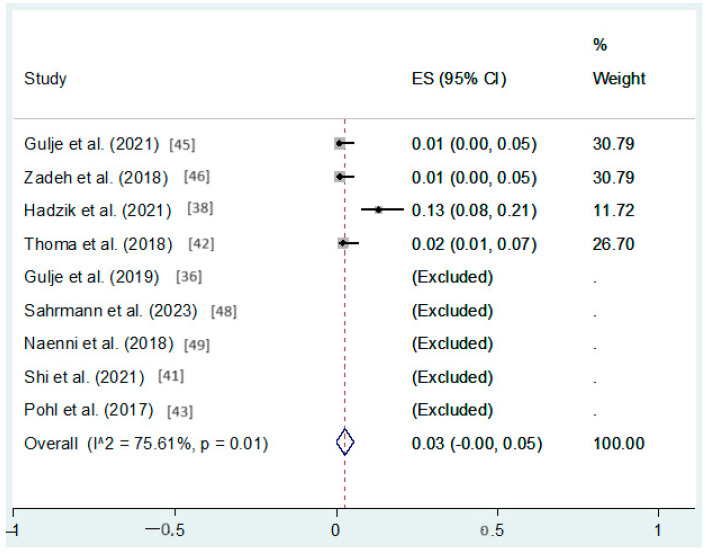
Forest plot applying random-effect meta-analysis, assessing the difference in peri-implantitis rates between the short and long groups (data available in N = 9 studies) [36,38,41,42,43,45,46,48,49]. In this forestplot, the box in the middle of each horizontal line (confidence interval, CI) represents the point estimate of the effect for a single study. The size of the box is proportional to the weight of the study in relation to the pooled estimate. The diamond represents the overall effect estimate of the meta-analysis. The dashed vertical line represents the line of no effect, with the value of 0 for continuous measure such as mean difference. The placement of the center of the diamond on the x-axis represents the point estimate, and the width of the diamond represents the 95%CI around the point estimate of the pooled effect.

Nine studies reported on the prevalence of peri-implantitis which ranged from 0% to 6% and 0% to 13% in the short and long implant groups, respectively. The combined random-effect analysis showed an increased, although not statistically significant, prevalence of peri-implantitis in the longer implants (95%CI: −0% to 5%, *p* > 0.05) (Figure 4).

### 3.9. Technical/Prosthetic Complications

Ten studies assessed technical/prosthetic complications, which included abutment screw loosening, loss of abutment, loosening of suprastructure, chipping of ceramics, displaced healing cap, and fracture of definitive restoration. Studies assessing technical complications at the implant and patient level, respectively, were grouped together and analyzed separately. One study found no technical complications in the short or long implant groups 5 years post-loading [45]. No statistically significant differences were observed between short and longer implants at both the implant and patient level in terms of technical complications (implant level 95%CI: −4% to 6%, *p* > 0.05, patient level 95%CI: −21% to 10%, *p* > 0.05, in both cases I^2^ > 34%) (Figure S16).

## 4. Discussion

In the past few decades, various studies have explored the use of short implants for the rehabilitation of resorbed jaws, as an alternative treatment option to bone augmentation and standard-size implant placement. Among the available systematic reviews on this topic, only a few focused on truly short implants (≤6 mm). In the last five years, several RCTs have been published comparing short and longer implants. The present systematic review includes more recent data and studies with longer follow-up periods than previous systematic reviews [4,10,11,54]. Moreover, all possible meta-analyses were performed, ensuring a robust assessment of data and enhancing the reliability of the findings.

The studies included in this review recruited systemically healthy individuals to eliminate any possible effect of the systemic condition on implant outcomes. Nonetheless, some studies recruited patients with risk factors for biological and technical complications, including a history of periodontitis, smoking, and bruxism. A history of periodontal disease is associated with a higher risk for peri-implantitis and lower implant survival and success rates [55,56]. Heavy smoking (>20 cigarettes/day) may predispose for greater MBL [57] and implant failure compared to non-smoking status [58], while bruxism may be linked to increased technical complications [59] and implant failure [60]. Studies recruiting patients with the aforementioned risk factors were included in this systematic review since these patients constitute a considerable part of the population and shall not be excluded from implant treatment, provided that they follow a strict long-term maintenance program.

The present systematic review presents the outcomes of 16 studies, among which 9 were original studies. Articles reporting on different follow-ups of the same original study were included in this review, because they provide valuable data concerning the different observation timepoints. These data were used in the sub-group analyses according to follow-up duration (1 year, 3 years, 5 or more years) for the evaluation of implant survival rate and marginal bone loss.

The results of the present meta-analysis indicate significantly increased survival rates in standard-length implants, placed in pristine or augmented bone, compared to short implants for follow-up periods from 1 to 10 years. Significant heterogeneity was found in the overall analysis and in the analysis of studies with a follow-up at least 5 years. Heterogeneity may be attributed to differences in study protocols which lead to variability in the survival rates among RCTs. In particular, the included studies present differences in terms of the short and standard implant length and diameter, loading protocol, implant surface modifications, augmentation procedure, and experience of the surgeon, among others. Papaspyridakos et al. compared short rough-surfaced implants to longer implants and indicated that the survival rates of short implants present a higher variability and lower predictability after 1 to 5 years [11]. A recently published systematic review comparing 6 mm implants in pristine bone and 8 mm implants in augmented bone found comparable survival rates at 1 and 3 year follow-up recalls and significantly increased survival rates for 8 mm implants 5 years after implant loading [13]. Nonetheless, other studies have suggested that short implants present comparable survival rates to longer implants [4,12,54,61]. One possible explanation for the divergence in our findings could be the inclusion of different, more recently published studies. Also, factors including a high crown-to-implant ratio in short implants, an increased likelihood of low-density bone in the posterior maxilla, and the anticipated accelerated progression of peri-implantitis around shorter implants should be considered when selecting short implants. Meanwhile, the predictability of different bone augmentation procedures in the maxilla or mandible and their potential complications should be carefully evaluated to select the more effective treatment plan for each patient. The predictability of vertical bone augmentation in the posterior mandible and in the posterior maxilla (sinus floor augmentation) differ. For that reason, we performed sub-group analysis comparing short implants in pristine bone and long implants in augmented bone in the posterior maxilla.

The findings of the present study suggest that increased MBL values are associated with longer implants at all follow-up periods, although the difference is not statistically significant. Analysis was also performed based on two different timepoints for MBL measurement (implant placement and implant loading) reported in the studies and confirmed the above result. No significant heterogeneity was present. An increased MBL for long implants was also observed when comparing short implants in pristine and long implants in augmented bone, as well as short and long implant placement in the posterior maxilla. MBL after implant placement and abutment connection is more pronounced, while bone alterations after implant loading are rather small [62]. Meanwhile, it is important to consider bone loss relative to implant length. In other words, the same absolute value of MBL could be more detrimental for a short rather than a long implant. Several systematic reviews indicate favorable results for short implants in comparison to longer implants placed in pristine [12] or augmented bone [13,54,63], with respect to MBL. Bone regeneration prior to implant placement and early implant loading have been associated with a higher MBL [13,54].

An important factor related to marginal bone stress [64] and MBL [65] is crown height, defined as the perpendicular distance between the occlusal plane and the bone crest. This is critical especially for the restoration of posterior jaws with short implants, due to high masticatory forces applied in these areas. Almost all RCTs included in this review reported a significantly higher crown-to-implant ratio for short implants because of the decreased length of short implants and the increased height of the suprastructure. Although some studies suggested that MBL is higher in implants with a low crown-to-implant ratio and vice versa [66,67,68], other studies indicated no such association [65,69,70,71].

To the best of our knowledge, no systematic review has performed meta-analysis concerning the prevalence of peri-implantitis in short (≤6 mm) and long (>6 mm) implants. The present review indicated no statistically significant difference in peri-implantitis rates between short and longer implants. Guida et al. observed no difference in biological complications (peri-implant mucositis and peri-implantitis) between short (≤6 mm) to longer (≥8.5 mm) implants in pristine bone [12]. It would be logical to assume that peri-implantitis is not related to implant length, since it has a distinct etiology. Nonetheless, bone loss attributed to peri-implantitis is more critical for a short implant and presents more rapid progression [63]; meanwhile resection surgery may not be indicated for the treatment of peri-implantitis around short implants [14]. It would be worth to mention that different criteria for the definition of peri-implantitis have been used among the included studies. Three studies defined peri-implantitis as implants presenting bleeding on probing or suppuration, and marginal bone loss at least 2 mm [36,38,42], while one other study additionally included probing depth > 5 mm as a criterion for the diagnosis of peri-implantitis [46]. Two studies adhered to the definition of peri-implantitis described by Berglundh et al. (2018) which included bleeding/suppuration on gentle probing, increased probing depth compared to previous examination, and progressive interproximal radiographic bone loss [41,45]. In the study by Sahrmann et al., peri-implantitis was characterized by peri-implant probing depths exceeding 4 mm, suppuration, or progressive marginal bone loss [48], while Naenni et al. included a pocket depth > 5 mm in combination with suppuration and/or progressive marginal bone loss [49].

No significant differences between short and long implants were observed in terms of technical complications. Several previous systematic reviews reached similar results [3,9,10], while others indicated increased complications at specific follow-up recalls in short implants [52,61]. The increased crown-to-implant ratio, commonly observed in short implants, may be a risk factor for prosthetic complications [10]. It is therefore suggested to splint prostheses involving adjacent short implants in order to reduce their resistance to rotational movements and increase their stability to eccentric forces [70]. Concerning the loading protocols, it has been reported that short implants with immediate or early loading may present satisfactory survival rates and reduced marginal bone loss [72]. It is also recommended to splint prosthetic restorations of adjacent short implants [10]. Concerning the type of prosthetic restoration, it has been reported that cemented restorations are associated with increased survival rates and lower MBL values than screwed prostheses [73].

Among the included studies, differences were noted in terms of the implant system used and the implants’ surface characteristics. All studies applied implants with a nano-structured surface; five original studies used implants with a TiO_2_-blasted fluoride-modified surface, and the remaining four studies used implants with a sand-blasted acid-etched surface. The later surface modification enhances the hydrophilicity of the implant surface and enhances osseointegration [74], while fluoride modification enhances rapid healing and allows for early loading [75]. A recent systematic review suggested that fluoride-modified and sand-blasted acid-etched short implants (≤8 mm) present an increased risk for failure compared to their long (>8 mm) counterparts [75]. Surface modification overall enhances the survival rate of implants, although overall the survival rate is also influenced by other factors, including the bone quality, loading, and crown-to-implant ratio [75]. Surface roughness has not been proved to prevent MBL [76,77] nor the onset of peri-implantitis [78], but patient-related factors such as a history of periodontitis and smoking are deemed to be more clinically important for MBL [79]. Surprisingly, recently, in a pre-clinical model, it has been suggested that acid-etched surfaces enhance the early formation of bone-to-implant contact [80].

Overall, clinical decision-making concerning dental implant placement in atrophic jaws includes the assessment of several factors. Anatomical considerations include the proximity of the area of interest to vital anatomical structures such as the mandibular canal, the mental foramen, and the sinus [54]. After considering the difficulty, predictability, and possible intraoperative and postoperative complications of the indicated bone augmentation procedure in the area of interest, the clinician may decide if it would be more beneficial to perform the augmentation procedure that would allow for standard-length implant placement or place a short implant [61]. Medically compromised patients or patients unwilling to undergo extensive bone augmentation procedures may be candidates for short implants [6]. However, when considering short implants for patients with single missing molars and parafunctional habits, the increased risk of occlusal overload should be carefully evaluated [10]. In fact, a recent systematic review yielded that the incidence of adverse effects and prosthetic failures was higher in non-splinted implants compared to splinted [81].

To minimize selection bias, we used comprehensive and objective inclusion criteria, ensuring that a wide range of relevant studies were considered. Two different databases were searched to capture a wide range of studies and the literature search was thoroughly designed. Additionally, the selection process was performed by two independent reviewers (R.E., X.D.), who were blinded to each other’s decisions. To adhere to the highest standard of evidence, only RCTs were incorporated in the current analysis, thus reinforcing the robustness of the conclusions. Additionally, most of the included studies used implants of similar diameter. Several long-term studies (≥5 years), which were lacking from previous reviews, were included in this paper, thus providing long-term data on short implants. Since the follow-up periods varied across the studies, we performed sub-group analyses according to follow-up duration (1 year, 3 years, 5 or more years). Nonetheless, the main limitation of the present systematic review is the variability between the studies regarding the sample size, patient profile, parafunctional habits, implant design, operator’s surgical technique, type of bone, loading protocol, timing, and type and retention of prosthetic restoration, as well as the definition of peri-implantitis. In other words, cofounding factors are not controlled. The aforementioned parameters may significantly impact data collection and comparisons and lead to heterogeneity in the meta-analysis. The adjustment of many of these parameters would lead to more comparable results and robust conclusions.

## 5. Conclusions

Short implants represent an acceptable alternative treatment option to standard-length implants for the rehabilitation of posterior jaws. Particularly in cases where bone augmentation procedures are associated with reduced predictability and increased morbidity and risk for complications, short implants appear to provide a promising alternative. However, they should be selected cautiously due to a potentially limited survival rate compared to implants longer than 6 mm. Future well-designed RCTs comparing the clinical and radiological results of short and long implants placed under similar conditions and including a thorough analysis of potential confounding factors are recommended. Studies comparing short and long implant placement in pristine bone and in the posterior mandible are especially limited.

## Figures and Tables

**Table 1:** Study characteristics of included studies on short implants.

Authors, Publication Year	Study Design and Follow-Up Period	Source of Recruitment (Number of Centers and Type)	Funding	Operator	Risk Factors 1. History of Periodontitis 2. Smokers Included 3. Bruxism	Implant Type (1-Piece/ 2-Piece)	Implant Surface Characteristics	Implant Location	Time of Implant Placement (Months)	Stages of Implant Placement	Healing Time before Implant Loading (Months)	Prosthetic Parameters 1. Restoration Type 2. Retention Method	Supportive Care for Implants
Gulje et al. (2021) [45]	5-year RCT	5 Universities, 1 private practice	Dentsply Sirona Implants	Single surgeon in each center	1. Yes 2. Yes † 3. Yes	OsseoSpeed, Astra Tech, 2-piece	Blasted fluoride-modified, nano-structured	Mx, Mn post	>4 months	One and two	1.5	1. FPD 2. Screw	N/a
Zadeh et al. (2018) [46]	3-year RCT												
Gulje et al. (2013) [50]	1-year RCT												
Gulje et al. (2019) [36]	5-year RCT	1 Private practice, 1 University	Dentsply Implants	N/a	1. N/a 2. Yes † 3. N/a	Dentsply Sirona, 2-piece	TiO_2_-blasted fluoride-modified surface, nano-structured	Mx post	N/a	Two	3	1. Single crowns 2. Cemented	Yearly
Hadzik et al. (2021) [38]	7-year follow-up study	1 University	Astra Tech, University Statutory	N/a	1. No 2. Yes † 3. No	Dentsply Sirona, 2-piece	Fluoride treated, nano-structured	Mx post	N/a	N/a	6	1. Single crown 2. Cemented	Yearly
Hadzik et al. (2018) [37]	3-year follow-up study												
Nielsen et al. (2021) [39]	1-year RCT	1 University hospital	Not applicable	N/a	1. Yes 2. Yes † 3. No	OsseoSpeed, Astra Tech	Blasted fluoride-modified, nano-structured	Mx post	>4 months	Two	7	1. Single crown 2. Screw	Biannually
Rokn et al. (2018) [35]	1-year RCT	1 University	Dental Implant Research Center, Dental Research Institute, Tehran University of Medical Sciences	N/a	1. N/a 2. N/a 3. N/a	Straumann, 2-piece	Sand-blasted large grit acid etched, nano-structured	Mn post	>6 months	One	2	1. Single crowns or FPD 2. Cemented	4 months after prosthetic loading and 1 year later
Rossi et al. (2016) [47]	5-year RCT	ITI Research Committee	“Clinics”	N/a	1. N/a 2. Yes 3. Yes	Straumann, 2-piece	Sand-blasted large grit acid-etched, nano-structured	Mx, Mn post	N/a	One	1,75	1. Single fixed prosthesis 2. N/a	N/a
Sahrmann et al. (2023) [48]	10-year RCT	1 University	ITI	Experienced surgeon	1. Yes 2. Yes † 3. No	Straumann, 2-piece	Sand-blasted large grit acid etched, nano-structured	Mx, Mn post	>6 months	One	2,5	1. Single crown 2. Screw	Annually
Naenni et al. (2018) [49]	5-year RCT												
Shi et al. (2021) [41]	3-year RCT	1 Hospital	ITI Foundation	Experienced surgeon	1. Yes 2. Yes † 3. N/a	Straumann, 2-piece	Sand-blasted large grit acid-etched, nano-structured	Mx post	>3 months	One	3	1. Single crowns, bridge 2. Cemented	Yearly
Shi et al. (2019) [40]	1-year RCT												
Thoma et al. (2018) [42]	5-year RCT	3 Universities, 1 academy, 1 private clinic	Dentsply Sirona Implants	N/a	1. N/a 2. Yes 3. Yes	Dentsply Sirona, 2-piece	Blasted fluoride- modified surface, nano-structured	Mx post	>4 months	One and two	6–7	1. Single crowns 2. Screw or cemented	“Regular maintenance”
Pohl et al. (2017) [43]	3-year RCT												
Schincaglia et al. (2015) [44]	1-year RCT												

RCT: randomized controlled trial, Mx: maxilla, Mn: mandible, post: posterior location, FPD: fixed partial denture, N/a: not available, †: heavy smokers excluded.

**Table 2:** Study characteristics and outcomes of the included studies on short implants.

Authors, Publication Year	Number of Patients per Group (Implants per Group)		Dropouts (Implants Number, If Available)		Mean age ± SD or Mean Age (Range), Years		Implant Characteristics 1. Implant Length 2. Implant Diameter		Surgical Parameters 1. Augmentation Performed 2. Surgical Intervention (Flapless/Flap)		Outcomes 1. Survival Rate (%) 2. Failure (Early, Late) 3. MBL, mm		Outcomes 1. Peri-Implantitis 2. Technical Complications 3. Other	
	Short	Long	Short	Long	Short	Long	Short	Long	Short	Long	Short	Long	Short	Long
Gulje et al. (2021) [45] 5-year RCT	49 (108)	46 (101)	3 (10)	6 (13)	55 ± 9 (26–69)	54 ±10 (34–70)	1. 6 2. 4	1. 11 2. 4	1. Autogenous grafting ^‡^ 2. Flap	1. Autogenous grafting ^‡^ 2. Flap	1. 96.0 ^#^ 2. 3; 1 3. −0.01 ± 0.45 ^#^	1. 98.9 2. 0; 1 3. 0.12 ± 0.93	1. 6% ^#^ 2. 12 3. c/I ratio: 1.78 ± 0.35 *	1. 7% 2. 18 3. 0.93 ± 0.17
Zadeh et al. (2018) [46] 3-year RCT	49 (108)	46 (101)	3 (10)	6 (13)	55 ± 9 (26–69)	54 ±10 (34–70)	1. 6 2. 4	1. 11 2. 4	1. Autogenous grafting ^‡^ 2. Flap	1. Autogenous grafting ^‡^ 2. Flap	1. 96.2 ^#^ 2. 3; 1 3. −0.04 ± 0.43 ***	1. 99 2. 0; 1 3. 0.02 ± 0.76	1. 0 2. 13 3. c/I ratio: 1.78 ***	1. 1.2% 2. 13 3. 0.93
Gulje et al. (2013) [50] 1-year RCT	49 (108)	46 (101)	1 (1)	0	55 ± 9 (26–69)	54 ±10 (34–70)	1. 6 2. 4	1. 11 2. 4	1. Autogenous grafting ^‡^ 2. Flap	1. Autogenous grafting ^‡^ 2. Flap	1. 97 2. 1; 11 3. −0.06 (SD 0.27) ^#^	1. 99 2. 0;1 3. 0.02 (SD 0.6)	1. N/a 2. 7	1. N/a 2. 8
Gulje et al. (2019) [36] 5-year RCT	20 (21)	18 (20)	1	1	50 (30–71)	48 (29–72)	1. 6 2. N/a	1. 11 2. N/a	1. No 2. Flap	1. Sinus floor augmentation 2. Flap	1. 94.7% ^#^ 2. 0;1 3. 0.12 ± 0.36 ^#^	1. 100% 2. 0; 0 3. 0.14 ± 0.63	1. 0 2. 4 patients ^#^	1. 0 2. 1 patient: screw loosening
Hadzik et al. (2021) [38] 7-year follow-up study	15 (15)	15 (15)	2 (2)	0	48.8 (26–64)	42.3 (26–63)	1. 6 2. 4	1. 11, 13 2. 4	1. No 2. N/a	1. Lateral sinus floor elevation 2. Flap	1. 87 ^#^ 2. 0;2 3. 0.5 ^#^	1. 100 2. 0; 0 3. 0.52	1. 0% 2. 2 3. C/I ratio:1.64 *	1. 13% 2. 3 3. 1.06
Hadzik et al. (2018) [37] 3-year follow-up study	15 (15)	15 (15)	N/a	N/a	48.8 (26–64)	42.3 (26–63)	1. 6 2. 4	1. 11, 13 2. 4	1. No 2. N/a	1. Lateral sinus floor elevation 2. Flap	1. 100 ^#^ 2. 0; 0 3. 0.22 ± 0.46 ^#^	1. 100 2. 0; 0 3. 0.34 ± 0.24	1. N/a 2. N/a	1. N/a 2. N/a
Nielsen et al. (2021) [39] 1-year RCT	20 (20)	20 (20)	0	3 (3)	52		1. 6 2. 4.2	1. 13 2. 4.2	1. No 2. Flap	1. Sinus floor augmentation 2. Flap	1. 100 2. 0; 0 3. 0.60 (SD 0.17) ^#^	1. 100 2. 0; 0 3. 0.51 (SD 0.14)	1. N/a 2. 2	1. N/a 2. 6
Rokn et al. (2018) [35] 1-year RCT	11 (25)	11 (22)	1	1	50.3		1. 4 2. 4.1	1. 8, 10 2. 4.1	1. No 2. Flap	1. Vertical bone augmentation 2. Flap	1. 100 2. 0; 0 3. 0.30 ± 0.34 ^#^	1. 100 2. 0; 0 3. 0.47 ± 0.54	1. N/a 2. N/a	1. N/a 2. N/a
Rossi et al. (2015) [47] 5-year RCT	30 (30)	30 (30)	0	0	48.4	47.7	1. 6 2. 4.1	1. 10 2. 4.1	1. No 2. Flap	1. No 2. Flap	1. 86.7 2. 1; 3 3. 0.14	1. 96.7 2. 0; 1 3. 0.18	1. N/a 2. 0 3. C/I ratio: 1.55	1. N/a 2. 0 3. 0.97
Sahrmann et al. (2023) [48] 10-year RCT	47 (47)	47 (47)	11 (11)	13 (13)	59.4 ± 11.3	61 ± 12.7	1. 6 2. 4	1. 11, 13 2. 4	1. No 2. N/a	1. Trans-crestal sinus lift ‡ 2. N/a	1. 85.7 ^#^ 2. 0; 6 3. 0.13 ^#^	1. 97.1 2. 0; 1 3. 0.08	1. 0 2. N/a 3. C/I ratio: 1.06 ± 0.18 ***	1. 0 2. N/a 3. 0.73 ± 0.17
Naenni et al. (2018) [49] 5-year RCT	47 (47)	47 (47)	7 (7)	1 (1)	58.2 at the time of recall		1. 6 2. 4.1	1. 10 2. 4.1	1. No 2. Flap	1. Trans-crestal sinus lift ‡ 2. Flap	1. 91 * 2. 0; 4 3. 0.29 ^#^	1. 100 2. 0; 0 3. 0.15	1. 0 2. N/a 3. C/I ratio: 1.75 ***	1. 0 2. N/a 3. 1.04
Shi et al. (2021) [41] 3-year RCT	75 (75)	8 mm: 75 (75), 10 mm: 75 (75)	8 (8)	8 mm: 13 (13), 10 mm: 5 (5)	40.2 ± 12.8	8 mm: 36.3 ± 12.6, 10 mm: 45.6 ± 11.8	1. 6 2. 4.1, 4.8	1. 8, 10 2. 3.3, 4.1, 4.8	1. No 2. Flap	1. Yes, osteotome sinus floor elevation 2. Flap	1. 91.8 * 2. 2; 4 3. 0.53 ± 0.35 ^#^	8 mm: 1. 97.08 2. 0; 1 3. 0.50 ± 0.30 10 mm: 1. 100 2. 0; 0 3. 0.53 ± 0.28	1. 2 patients 2. a. Veneer chipping: 4 patients b. Loss of retention: 0	8 mm: 1. 2 patients 2. a. 4 patients b. 1 patient 10 mm: 1. 1 patient 2. a. 6 patients b. 0
Shi et al. (2019) [40] 1-year RCT	75 (75)	8 mm: 75 (75), 10 mm: 75 (75)	1 (1)	8 mm: 5 (5), 10 mm: 2 (2)	38.1	8 mm: 39.2 10 mm: 44.5	1. 6 2. 4.1, 4.8	1. 8, 10 2. 3.3, 4.1, 4.8	1. No 2. Flap	1. Yes, osteotome sinus floor elevation 2. Flap	1. 96 2. 2;1 3. 0.51 ^#^	8 mm: 1. 100 2. 0; 0 3. 0.47 10 mm: 1. 100 2. 0; 0 3. 0.52	1. N/a 2. N/a	1. N/a 2. N/a
Thoma et al. (2018) [42] 5-year RCT	50 (67)	51 (70)	6 (7)	5 (6)	50 ^#^ (23–76)	51 (20–77)	1. 6 2. 4	1. 11, 13, 15 2. 4	1. No 2. Flap	1. Lateral window sinus floor elevation 2. Flap	1. 98.5^#^ 2. 0;1 3. 0.12 ± 0.54 ^#^	1. 100 2. 0; 0 3. 0.18 ± 0.96	1. 0% ^#^ (PL) 2. 21 events 47.7% ^#^ (PL) 3. c/I ratio: 1.86 ± 0.23 **	1. 2% 2. 14 events 30.4% 3. 0.99 ± 0.17
Pohl et al. (2017) [43] 3-year RCT	50 (67)	51 (70)	5 (6)	2 (2)	50 ^#^ (23–76)	51 (20–77)	1. 6 2. 4	1. 11, 13, 15 2. 4	1. No 2. Flap	1. Lateral window sinus floor elevation 2. Flap	1. 100 2. 0; 0 3. a. 0.44 ± 0.56 ^#^ b. 0.1 ± 0.54 ^#^	1. 100 2. 0; 0 3. a. 0.43 ± 0.58 b. 0.25 ± 0.58	1. 0 2. 10 events ^#^ 3. c/I ratio: 1.86 ± 0.2	1. 0 2. 3 events 3. 0.99 ± 0.17
Schincaglia et al. (2015) [44] 1-year RCT	50 (67)	51 (70)	3 (4)	1 (1)	50 ^#^ (23–76)	51 (20–77)	1. 6 2. 4	1. 11, 13, 15 2. 4	1. No 2. Flap	1. Lateral window sinus floor elevation 2. Flap	1. 100 2. 0; 0 3.0.22 ± 0.3 ***	1. 100 2. 0; 0 3. 0.37 ± 0.59	1. N/a 2. N/a 3. c/I ratio: 1.86 ± 0.23 ***	1. N/a 2. N/a 3. 0.99 ± 0.17

SD: standard deviation, N/a: not available, Mx: maxilla, Mn: mandible, MBL: marginal bone loss until the last follow-up, c/I ratio: crown-to-implant ratio, PL: patient level, ‡: performed only when necessary, not in all cases, # indicates no statistical significance reported, * indicates statistical significance with *p* ≤ 0.05, ** indicates statistical significance with *p* ≤ 0.01, *** indicates statistical significance with *p* ≤ 0.001.

**Table 3:** Details of risk of bias assessment performed for randomized controlled trials.

	D1	D2	D3	D4	D5	Overall		
Gulje 2021 [45], Zadeh 2018 [46], Gulje 2013 [50]	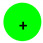	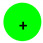	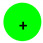	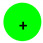	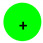	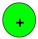	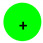	Low risk
Gulje 2019 [36]	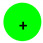	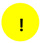	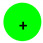	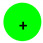	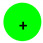	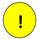	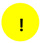	Some concerns
Hadzik 2021 [38], Hadzik 2018 [37]	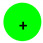	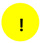	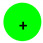	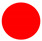	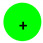	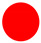	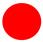	High risk
Nielsen 2021 [39]	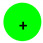	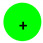	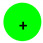	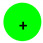	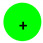	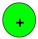		
Rokn 2018 [35]	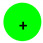	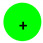	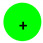	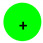	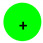	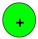	D1	Randomisation process
Rossi 2016 [47]	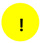	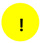	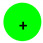	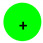	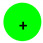	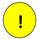	D2	Deviations from the intended interventions
Shi 2021 [41], Shi 2019 [40]	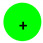	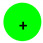	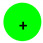	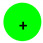	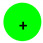	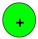	D3	Missing outcome data
Sahrmann 2023 [48], Naenni 2018 [49]	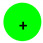	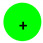	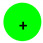	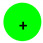	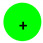	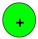	D4	Measurement of the outcome
Thoma 2018 [42], Pohl 2017 [43], Schincaglia 2015 [44]	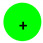	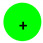	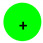	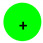	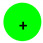	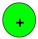	D5	Selection of the reported result

## Data Availability

The original contributions presented in the study are included in the article/supplementary material, further inquiries can be directed to the corresponding author.

## Supplementary Materials

The following supporting information can be downloaded at: https://www.mdpi.com/article/10.3390/dj12060185/s1, Figure S1: Summary plot for risk of bias of original RCTs included in this systematic review, Figure S2: Funnel plot of the 16 randomized clinical trials included in the present meta-analysis, Figure S3: Egger test taking into account the 16 randomized clinical trials included in the present meta-analysis, Figure S4: Forest plot applying fixed-effect meta-analysis, assessing the difference in survival rates between short and long groups (N= 16 studies), Figure S5: Forest plot applying random-effect meta-analysis, assessing the difference in survival rates between short and long groups (N= 16 studies), Figure S6: Forest plot applying random-effect meta-analysis, assessing the risk difference in early implant failure between short and long groups, according to follow-up period (N= 16 studies), Figure S7: Forest plot applying random-effect meta-analysis, assessing the risk difference in late implant failure between short and long groups, according to follow-up period (N= 16 studies), Figure S8: Forest plot applying random-effect meta-analysis, assessing the difference in survival rates between short and long groups, according to augmented or pristine bone (N= 16 studies), Figure S9: Forest plot applying random-effect meta-analysis, assessing the difference in survival rates between short and long groups, according to surgical parameters (N= 10 studies), Figure S10: Forest plot applying random-effect meta-analysis, assessing the difference in survival rates between short and long groups, to implant location-posterior maxilla and by follow-up period (N= 9 studies), Figure S11: Forest plot applying fixed-effect meta-analysis, assessing the mean difference in MBL (mm) between short and long groups (N= 16 studies), Figure S12: Forest plot applying random-effect meta-analysis, assessing the mean difference in MBL (mm) between short and long groups (N= 16 studies), Figure S13: Forest plot applying random-effect meta-analysis, assessing the mean difference in MBL (mm) between short and long groups, by healing time (N= 16 studies). Figure S14: Forest plot applying random-effect meta-analysis, assessing the mean difference in MBL (mm) between short and long groups according to surgical parameters (N= 10 studies), Figure S15: Forest plot applying random-effect meta-analysis, assessing the mean difference in MBL (mm) between short and long groups according to implant location- posterior maxilla and by follow-up period (N= 9 studies), Figure S16: Forest plot applying random-effect meta-analysis, assessing the risk difference of technical complications between short and long groups (data available in N= 9 studies), Figure S17: Forest plot applying random-effect meta-analysis, assessing the mean value in MBL (mm) in short group and by follow-up period, Figure S18: Forest plot applying random-effect meta-analysis, assessing the mean value in MBL (mm) in long group and by follow-up period, Figure S19: Forest plot applying random-effect meta-analysis, assessing the survival rate in short group and by follow-up period, Figure S20: Forest plot applying random-effect meta-analysis, assessing the survival rate in long group and by follow-up period, Table S1: Randomized clinical trials included in the present meta-analysis, regarding survival rate (%) difference between short and long group, Table S2: Results from fixed-effect meta-analysis, regarding survival rate (%) value difference between short and long groups, Table S3: Randomized clinical trials included in the present meta-analysis, regarding MBL (mm) mean value difference between short and long group.

## Appendix A. Details on the Search Strategies Applied

MEDLINE

((((((dental) OR (oral)) OR (osseointegrated)) AND (implant*)) AND (short)) NOT (review)) NOT (animal)

Filters: English

Sort by: Most recent

OVID

1. dental implant* .mp. [mp=title, abstract, full text, caption text]

2. oral implant* .mp. [mp=title, abstract, full text, caption text]

3. osseointegrated implant* .mp. [mp=title, abstract, full text, caption text]

4. 1 or 2 or 3

5. short implant* .mp. [mp=title, abstract, full text, caption text]

6. 4 and 5
