# Supplementary material for: Survival Rates of Short Dental Implants (≤6 mm) Used as an Alternative to Longer (>6 mm) Implants for the Rehabilitation of Posterior Partial Edentulism: A Systematic Review of RCTs
Source: Dent J (Basel). 2024 Jun 17;12(6):185. doi: 10.3390/dj12060185 (PMC11202938; doi:10.3390/dj12060185)
Supplement: Supplementary file 1 [file dentistry-12-00185-s001.zip › dentistry-2957722-supplementary.pdf]

Supplementary Material.

Figure S1

Summary plot for risk of bias of original RCTs included in this systematic review

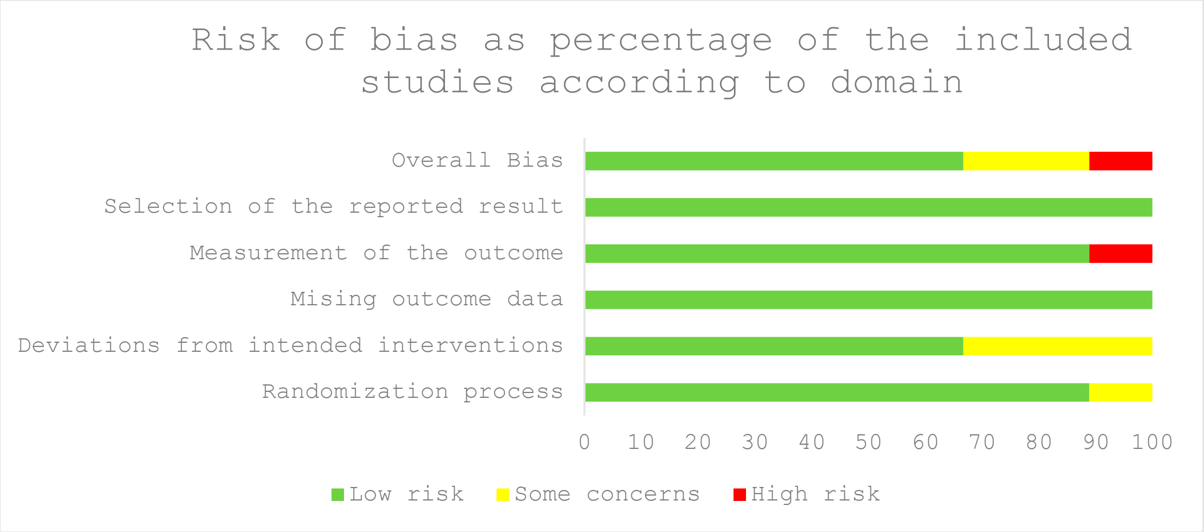

Figure S2 Funnel plot of the 16 randomized clinical trials included in the present meta-analysis.

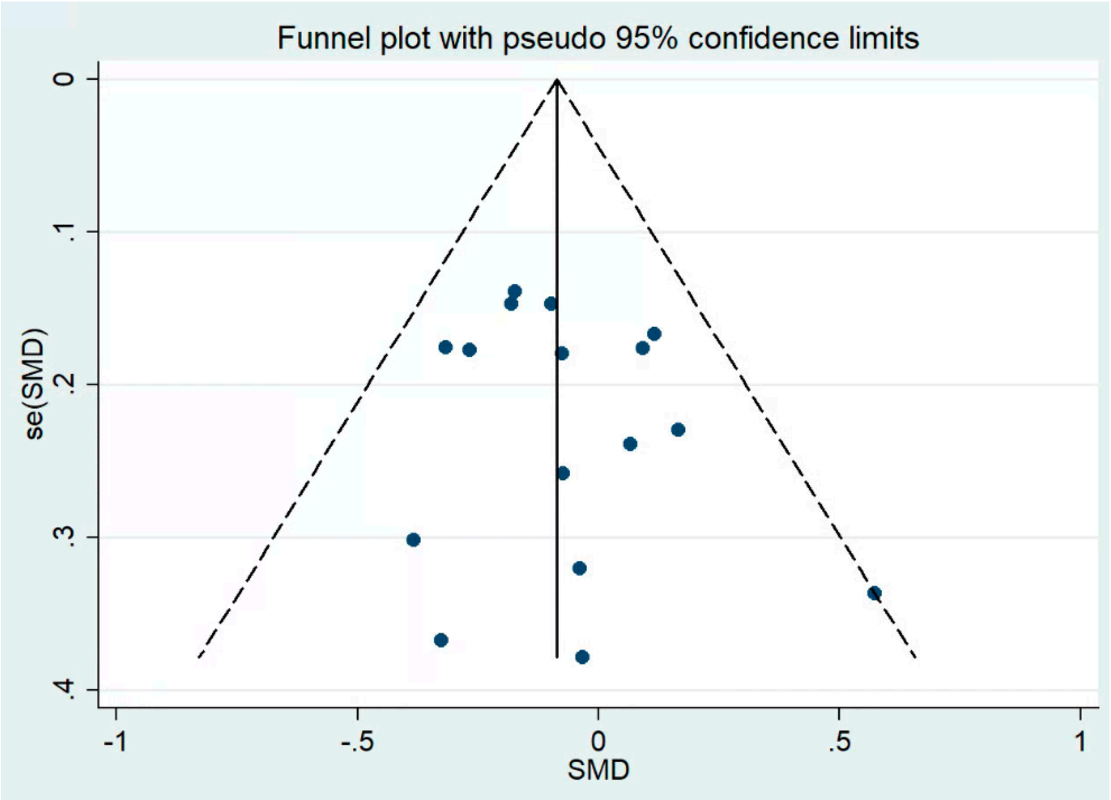

Figure S3. Egger test taking into account the 16 randomized clinical trials included in the present meta-analysis.

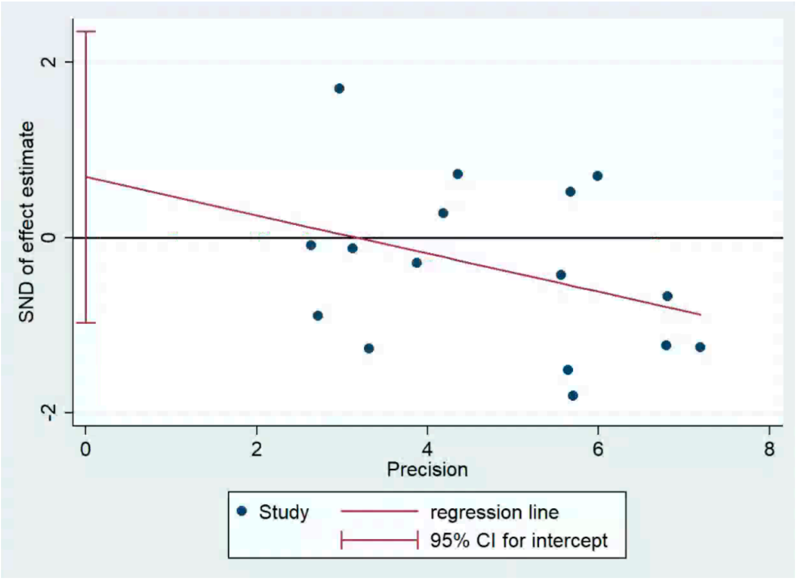

Figure S4. Forest plot applying fixed-effect meta-analysis, assessing the difference in survival rates between short and long groups (N= 16 studies).

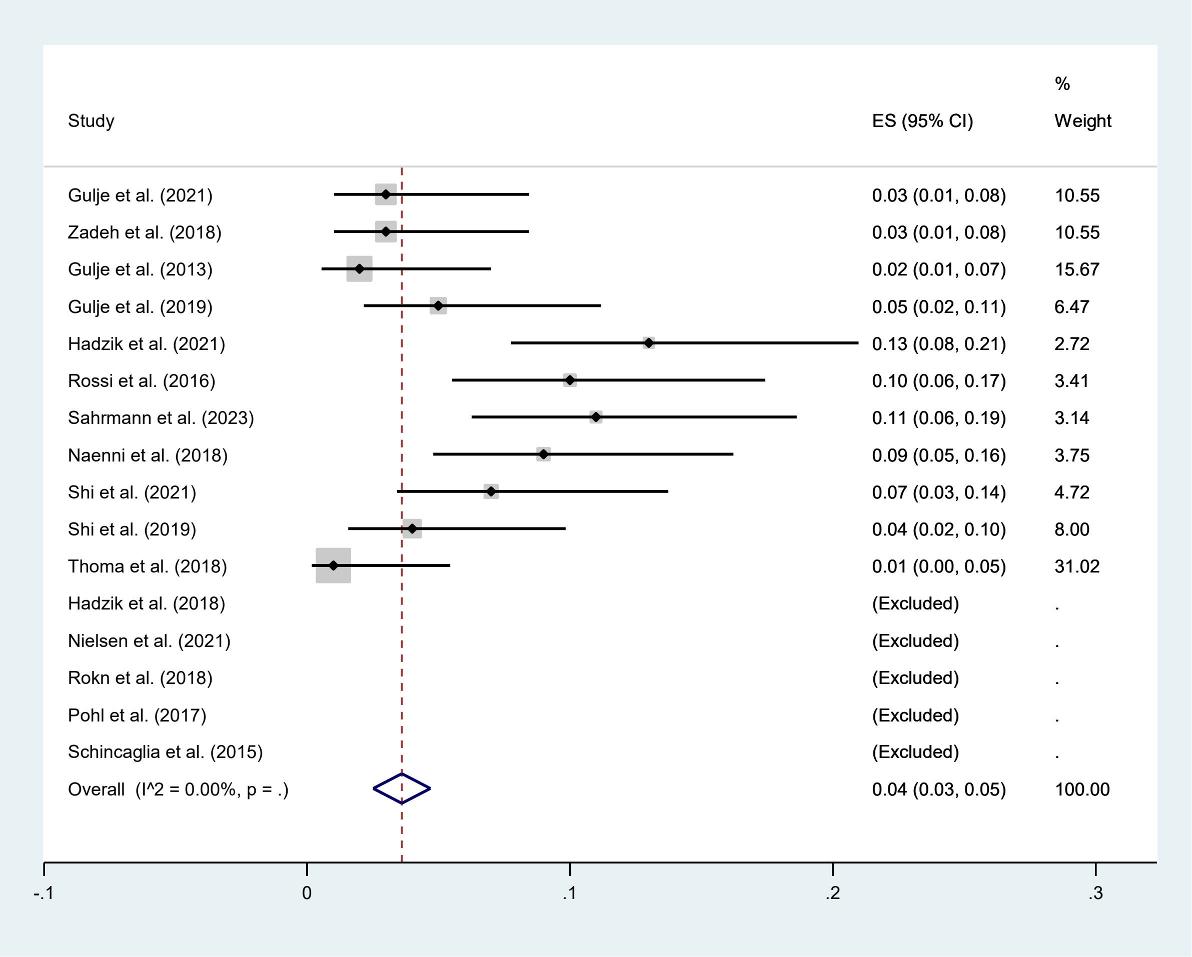

The combined fixed-effect shows that the total survival rate difference between short and long groups is associated with an increased survival rate in the long group (% difference: 3% and corresponding 95% Confidence Interval - CI (2% to 5%, z= 6.51 and p< 0.001). The result is statistically non-significant (Figure S4).

Figure S5. Forest plot applying random-effect meta-analysis, assessing the difference in survival rates between short and long groups (N= 16 studies).

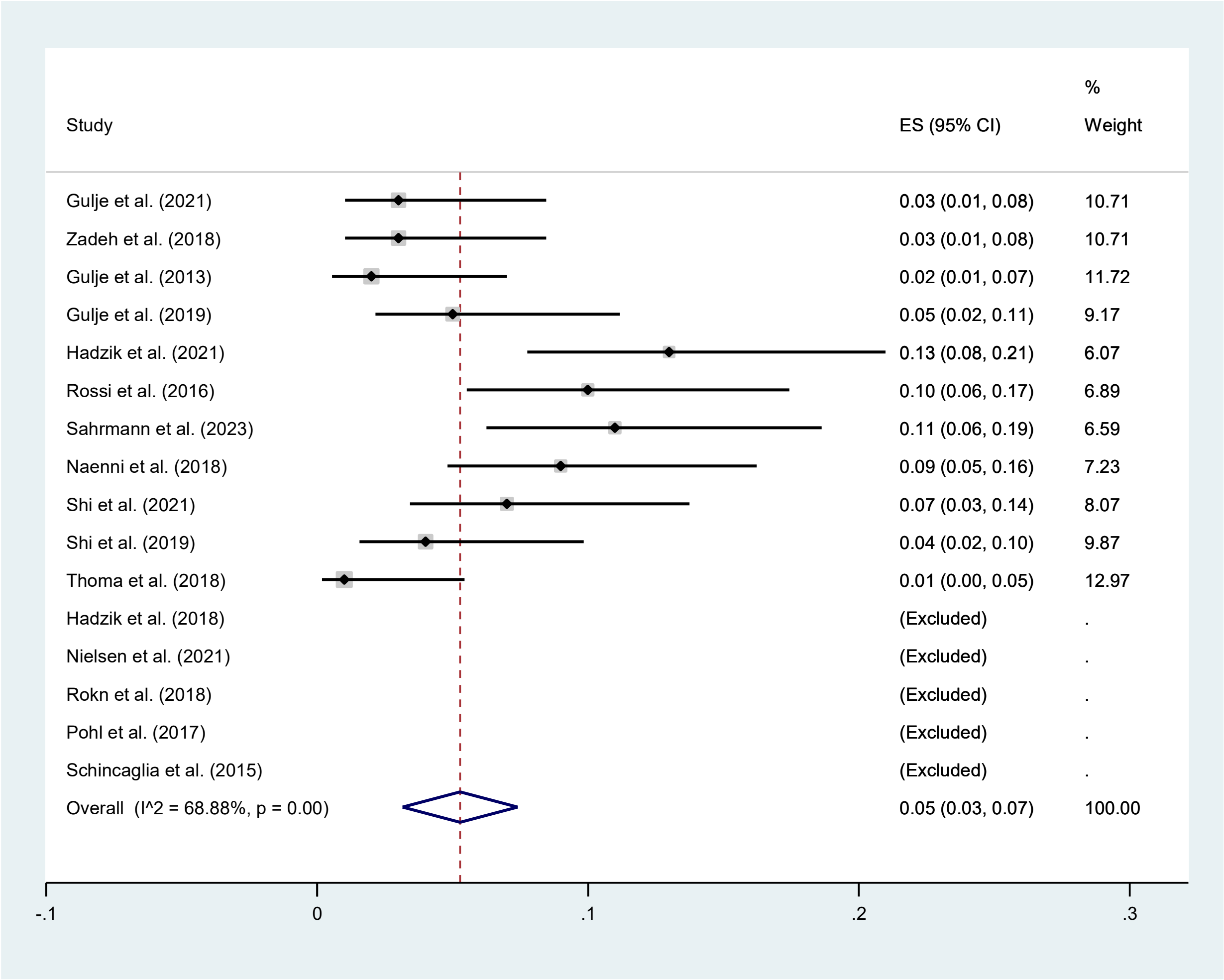

Figure S5 presents the combined random-effect result. Significant heterogeneity is present: $I^2 = 62.81\%$ and p-value<0.001. Therefore, the combined random-effect shows that the total survival rate difference between short and long groups is associated with an increased survival rate in the long group (% difference: 5% and corresponding 95% Confidence Interval - CI (3% to 7%, z= 4.89 and p< 0.001). Note that studies with a difference of 0 in survival rates are automatically excluded from the meta-analysis.

Figure S6. Forest plot applying random-effect meta-analysis, assessing the risk difference in early implant failure between short and long groups, according to follow-up period (N= 16 studies).

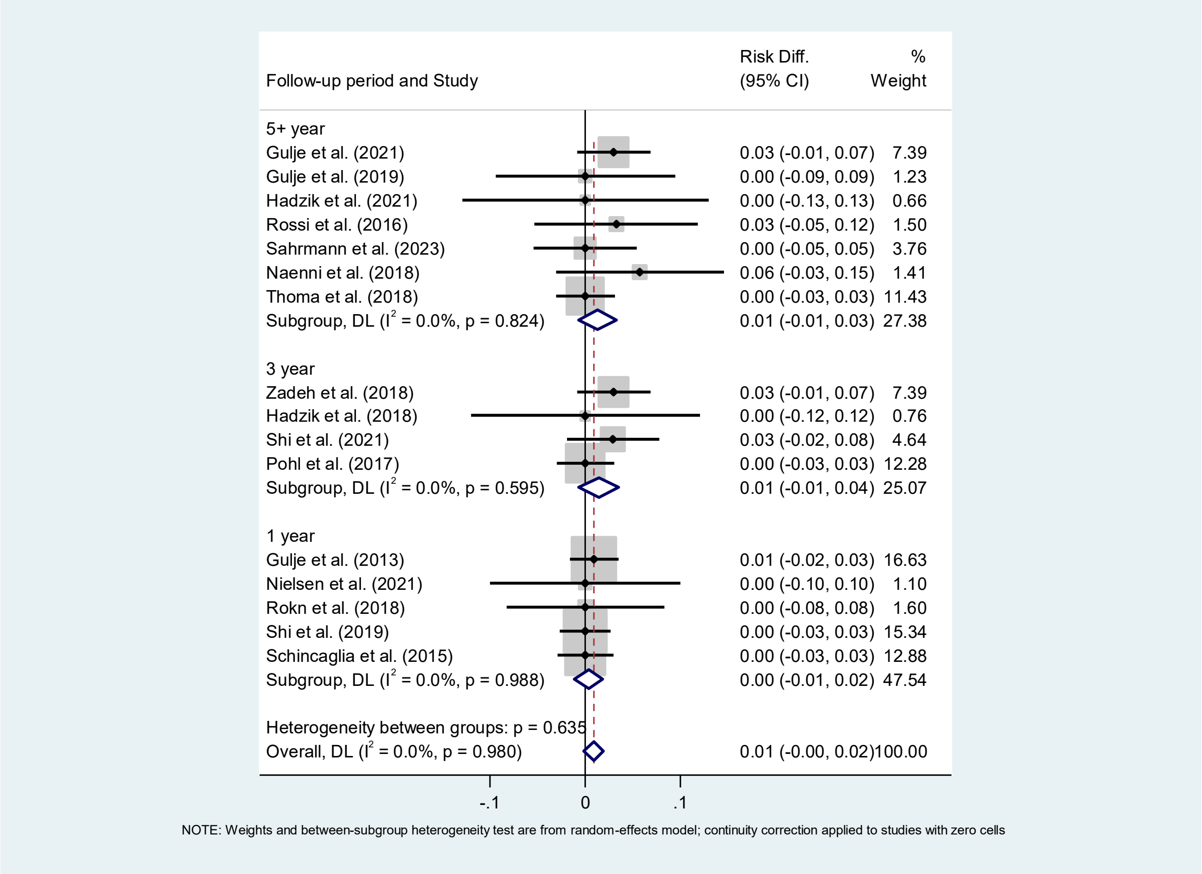

Figure S6 presents the combined random-effect risk difference in early implant failure between short and long groups, stratified by follow-up period. No significant heterogeneity is present: $I^2 = 0\%$ and p-values> 0.05. The overall pooled estimate shows that no risk difference regarding early implant failure between short and long groups is present (% difference: 0% and 95% CI (-1% to 2%, z= 1.601, p-value> 0.05). The combined effect for the 1,3 and 5+ years study period shows that the risk difference between short and long groups does not differ statistically significant (% difference: 0%, 1% and 1% with corresponding 95% CIs (-1% to 2%, z= 0.417; -1% to 4%, z= 1.321 and -1% to 3%, z= 1.245 respectively; p-values> 0.05).

Figure S7. Forest plot applying random-effect meta-analysis, assessing the risk difference in late implant failure between short and long groups, according to follow-up period (N= 16 studies).

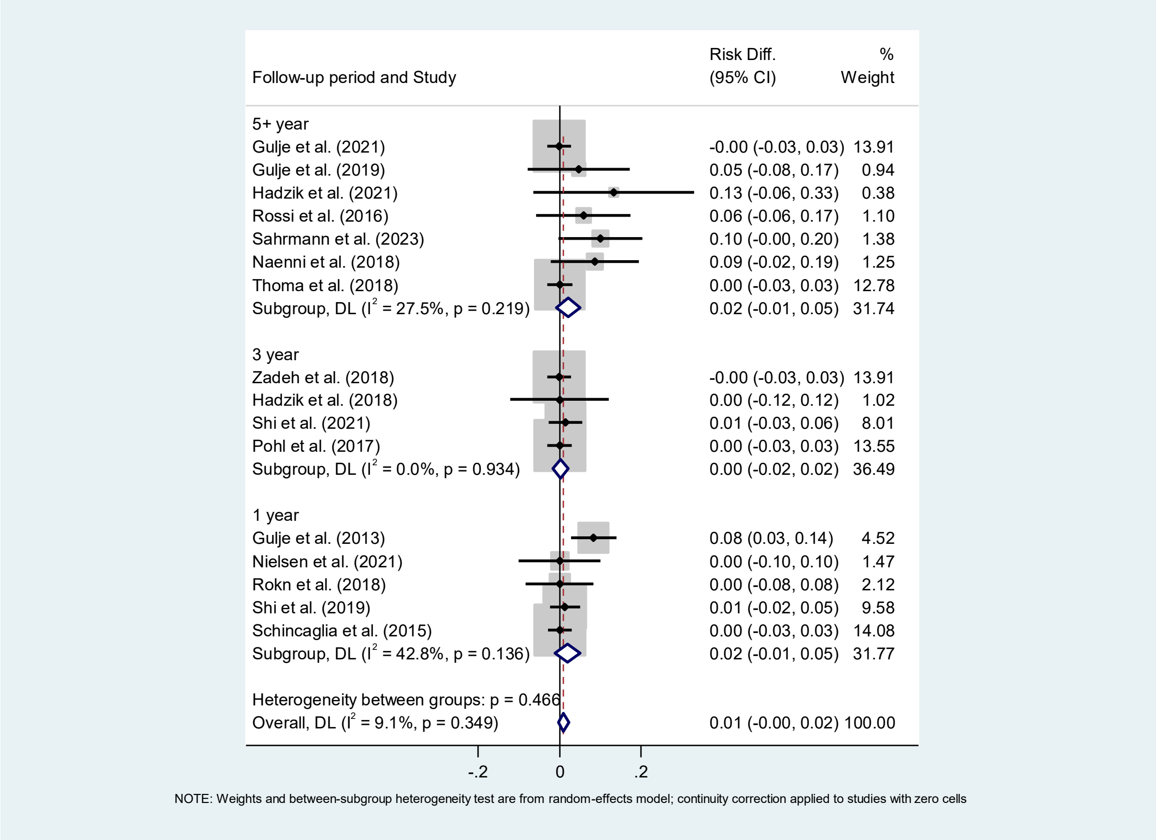

Figure S7 presents the combined random-effect risk difference in late implant failure between short and long groups, stratified by follow-up period. No significant heterogeneity is present: I^2 = <42% and p-values> 0.05. The overall pooled estimate shows that no risk difference related to late implant failure between short and long groups is present (% difference: 0% and 95% CI (-1% to 2%, z= 1.594, p-value> 0.05). The combined effect for the 1,3 and 5+ years study period shows that the risk difference between short and long groups does not differ statistically significant (% difference: 2%, 0% and 2% with corresponding 95% CIs (-1% to 5%, z= 1.252; -2% to 2%, z= 0.272 and -1% to 5%, z= 1.400 respectively; p-values> 0.05).

Figure S8. Forest plot applying random-effect meta-analysis, assessing the difference in survival rates between short and long groups, according to augmented or pristine bone (N= 16 studies).

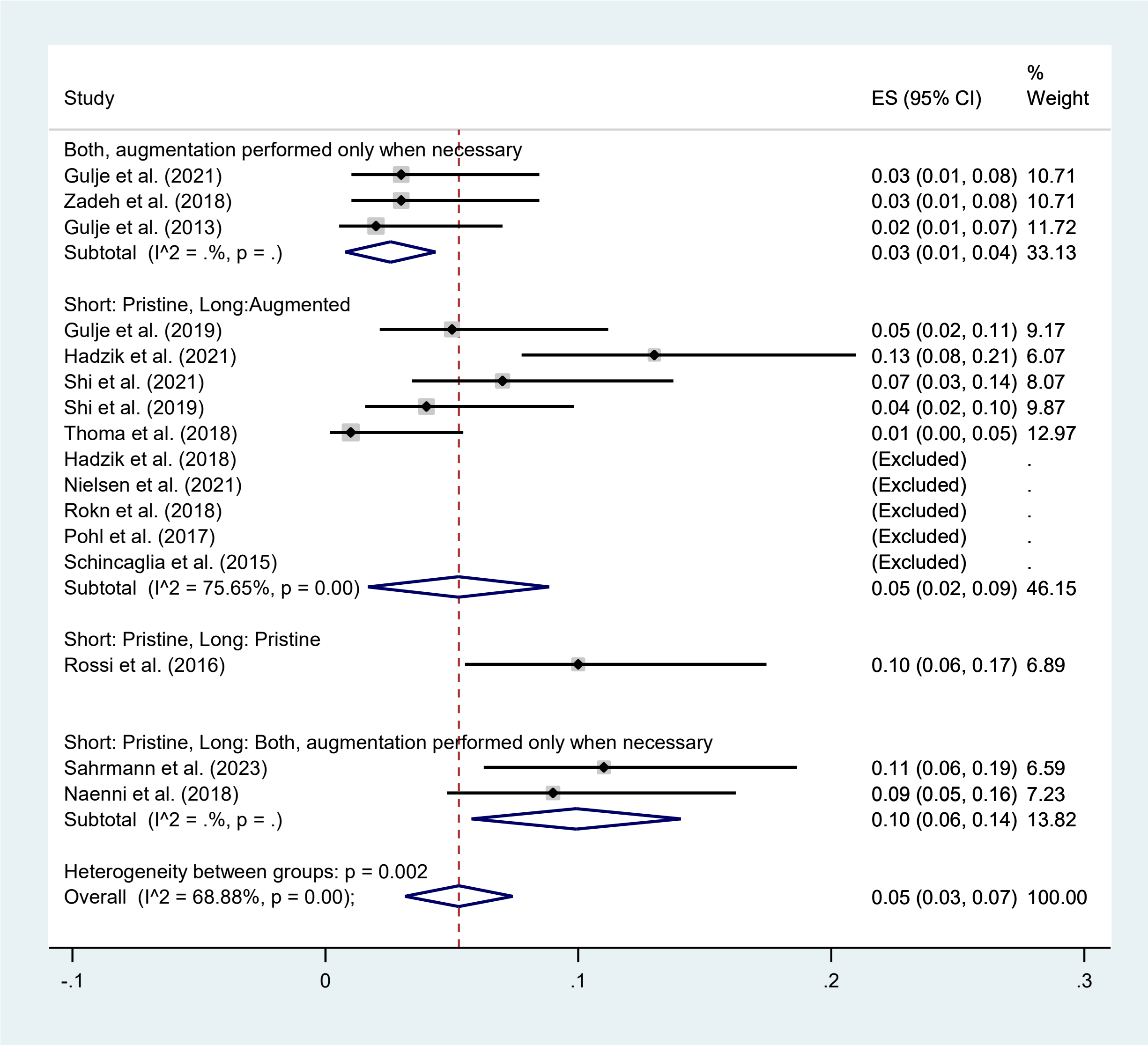

Figure S8 presents the combined random-effect estimates, according to augmented or pristine bone. Significant heterogeneity is present in subgroup studies with short group-pristine bone vs long group-augmented bone: I^2 = 75.66% and p-value<0.001. The aforementioned subtotal pooled estimate shows that the total survival rate difference between short and long groups is significantly associated with an increased survival rate in the long group (% difference: 5% and 95% CI (2% to 9%, z= 2.88, p-value< 0.05). The subtotal combined estimate for both, augmentation performed only when necessary shows that the total survival rate difference between short and long groups is significantly associated with an increased survival rate in the long group (% difference: 3% and 95% CI (1% to 4%, z= 2.82, p-value< 0.05). The subtotal combined estimate for short group-pristine bone vs long group-both, augmentation performed only when necessary shows that the total survival rate difference between short and long groups

is significantly associated with an increased survival rate in the long group (% difference: 10% and 95% CI (6% to 14%, z= 4.69, p-value< 0.05).

Figure S9. Forest plot applying random-effect meta-analysis, assessing the difference in survival rates between short and long groups, according to surgical parameters (N= 10 studies).

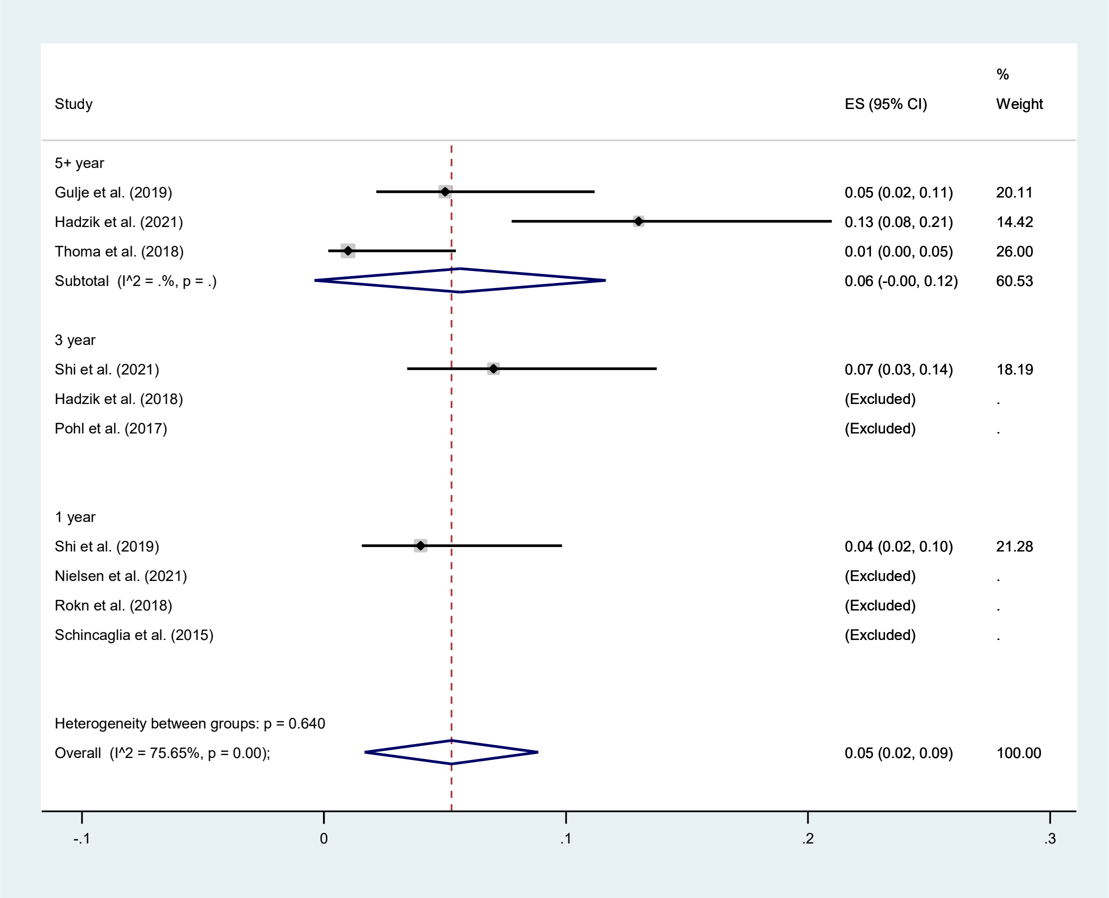

Figure S9 presents the combined random-effect estimates, according to surgical parameters and stratified by follow-up period. Significant heterogeneity is present: I^2 = 75.66% and p-value<0.001. The overall pooled estimate shows that the total survival rate difference between short and long groups is significantly associated with an increased survival rate in the long group (% difference: 5% and 95% CI (2% to 9%, z= 2.88, p-value< 0.05). The combined effect for 5+ years study period shows that the total survival rate difference between short and long groups is associated with an increased survival rate in the long group (% difference: 6% and 95% C (-0% to 12%, z= 1.83; p-value> 0.05), however a non-significant result.

Figure S10. Forest plot applying random-effect meta-analysis, assessing the difference in survival rates between short and long groups, to implant location-posterior maxilla and by follow-up period (N= 9 studies).

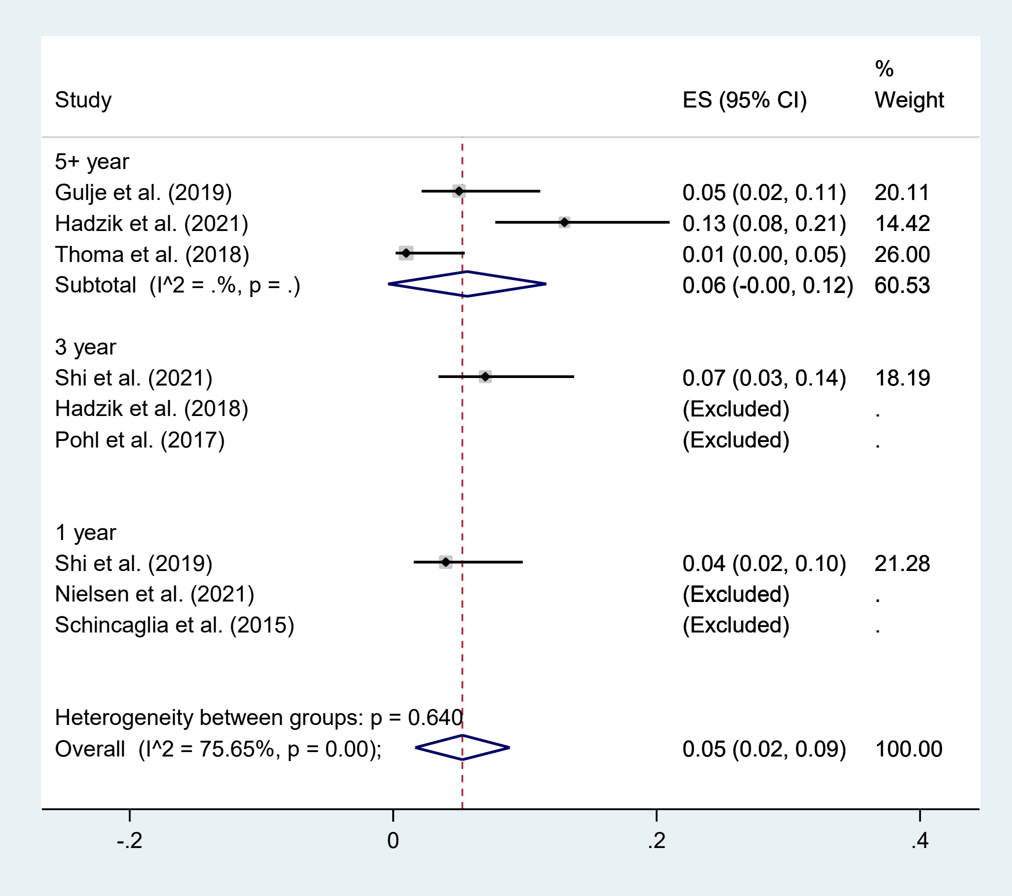

Figure S10 presents the combined random-effect estimates, according to implant location-post Mx and stratified by follow-up period. Significant heterogeneity is present: $I^2 = 75.66\%$ and p-value<0.001. The overall pooled estimate shows that the total survival rate difference between short and long groups is significantly associated with an increased survival rate in the long group (% difference: 5% and 95% CI (2% to 9%, z= 2.88, p-value< 0.05). The combined effect for 5+ years study period shows that the total survival rate difference between short and long groups is associated with an increased survival rate in the long group (% difference: 6% and 95% CI (-0% to 12%, z= 1.83; p-value> 0.05), however a non-significant result.

Figure S11. Forest plot applying fixed-effect meta-analysis, assessing the mean difference in MBL (mm) between short and long groups (N= 16 studies).

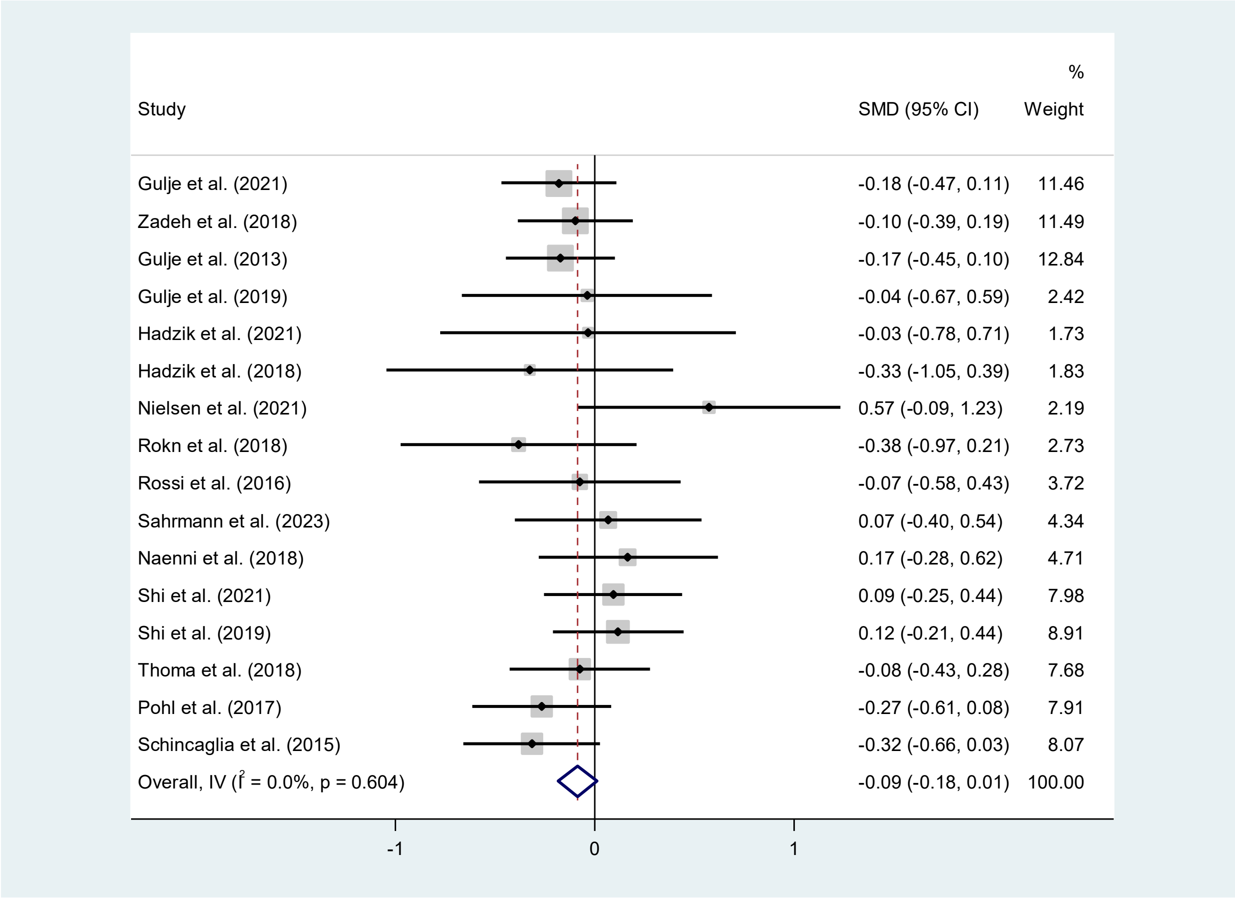

The combined effect shows that the total mean difference in MBL (mm) between short and long groups was associated with a reduced mean difference in MBL in short implants (standardized mean difference – SMD: -0,09 and corresponding 95% Confidence Interval - CI (-0.18 to 0.01, z= 1.74 and p= 0.082> 0.05). However, the result is statistically non-significant.

Figure S12. Forest plot applying random-effect meta-analysis, assessing the mean difference in MBL (mm) between short and long groups (N= 16 studies).

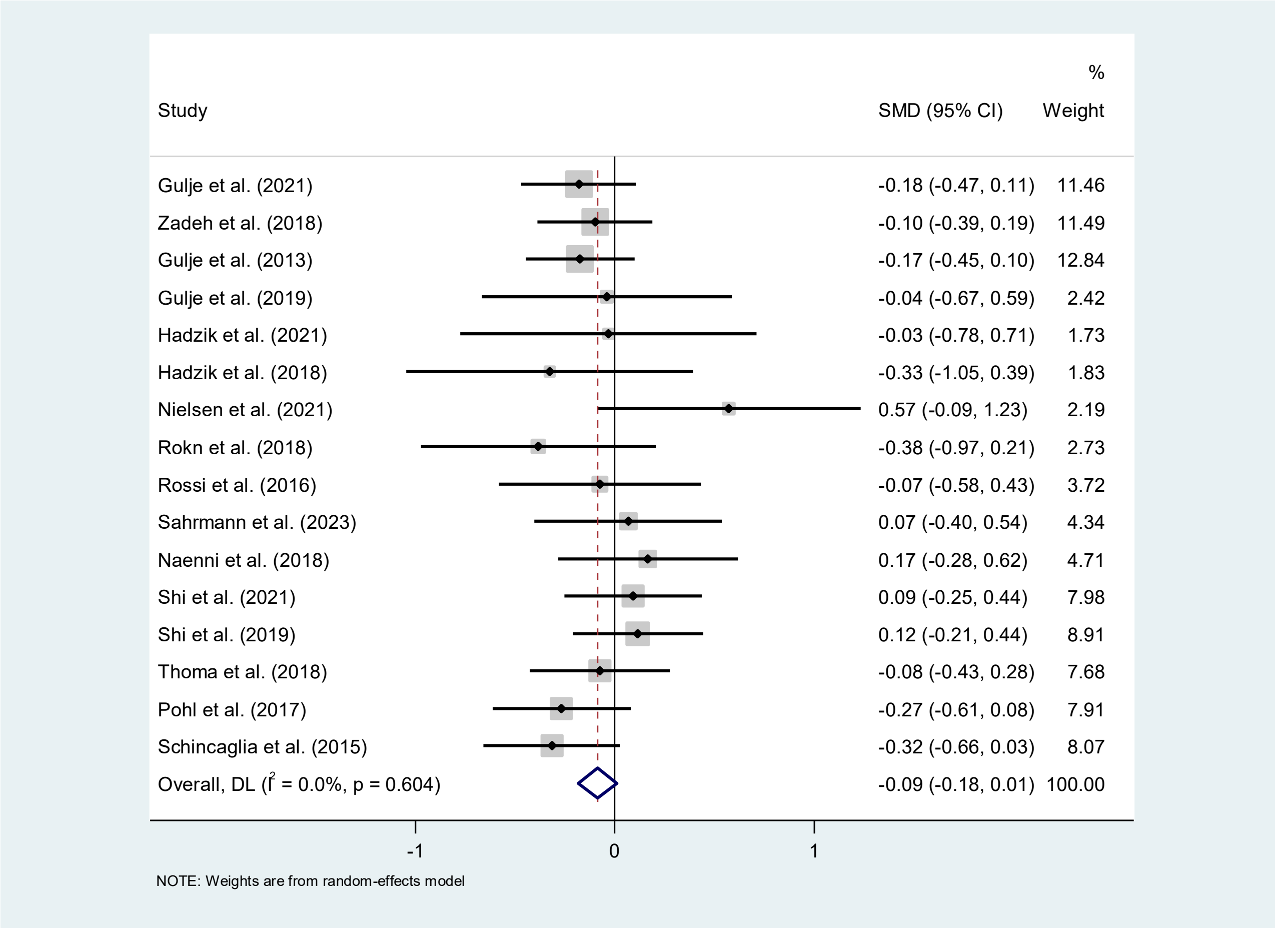

Figure S13. Forest plot applying random-effect meta-analysis, assessing the mean difference in MBL (mm) between short and long groups, by healing time (N= 16 studies).

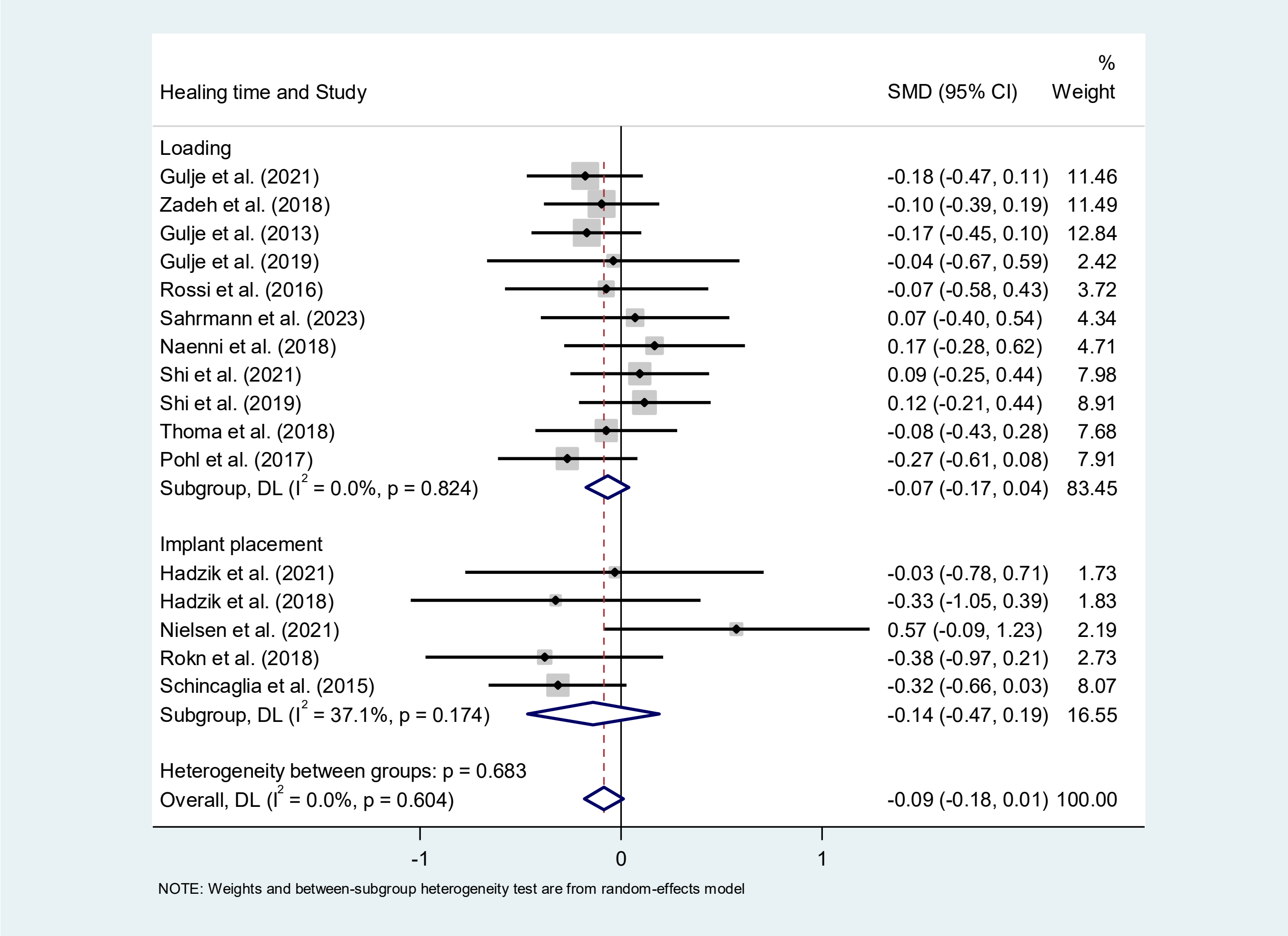

Figure S13 presents the combined random-effect of the mean difference in MBL (mm) between short and long groups, according to healing time including in total 16 studies. The combined effect for both loading and implant placement shows that the total mean difference in MBL (mm) between short and long groups was associated with a reduced mean difference in MBL in short implants (SMDs: -0,07 and -0.14 with corresponding 95% CIs (-0.17 to 0.04, z= -1.244 and -0.47 to 0.19, z= -0.836; p-values> 0.05). However, the results are statistically non-significant. No significant heterogeneity is present: range of I^2 = 0% and 37.1%; p-values> 0.05).

Figure S14. Forest plot applying random-effect meta-analysis, assessing the mean difference in MBL (mm) between short and long groups according to surgical parameters (N= 10 studies).

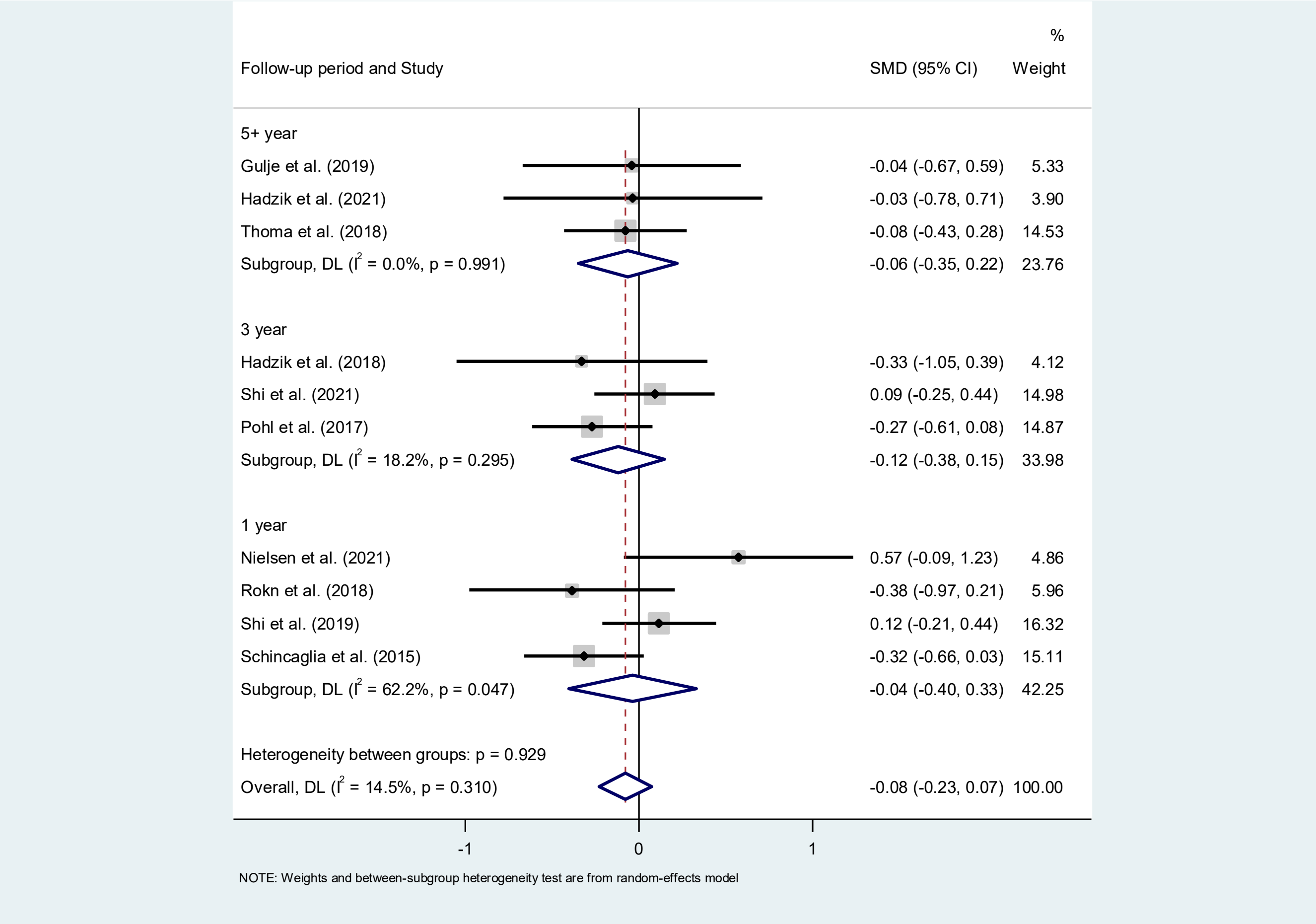

Figure S14 presents the combined random-effect of the mean difference in MBL (mm) between short and long groups, according to surgical parameters. In total, 10 studies were included in the present meta-analysis. Moreover, meta-analysis was performed by follow-up period. The overall combined effect shows that the total mean difference in MBL (mm) between short and long groups was associated with a reduced mean difference in MBL in short implants (SMD: -0,08 95% CI (-0.23 to 0.07, z= -1.008, p-value= 0.929> 0.05), however non-significant. No significant heterogeneity is present in the overall effect: I^2 = 14.5%, p-value= 0.310> 0.05). Following, the combined effect for each of the three follow-up periods: 1, 3, 5+ years shows that the total mean difference in MBL (mm) between short and long groups was associated with a reduced mean difference in MBL in short implants (SMDs: -0,04, -0.12 and -0.06 with corresponding 95% CIs (-0.66 to 0.03, z= -0.888; -0.38 to 0.15, z= -0.866; -0.35 to 0.22, z= -0.675, respectively; all p-values> 0.05). However, the results are statistically non-significant. No significant heterogeneity is present in the follow-up period of 3 and 5+years: I^2 = 0% to 18.2%; p-values> 0.05), in contrast to the studies with 1 year follow-up in which significant heterogeneity is present: I^2 = 62.2% and p-value= 0.047< 0.05.

Figure S15. Forest plot applying random-effect meta-analysis, assessing the mean difference in MBL (mm) between short and long groups according to implant location- posterior maxilla and by follow-up period (N= 9 studies).

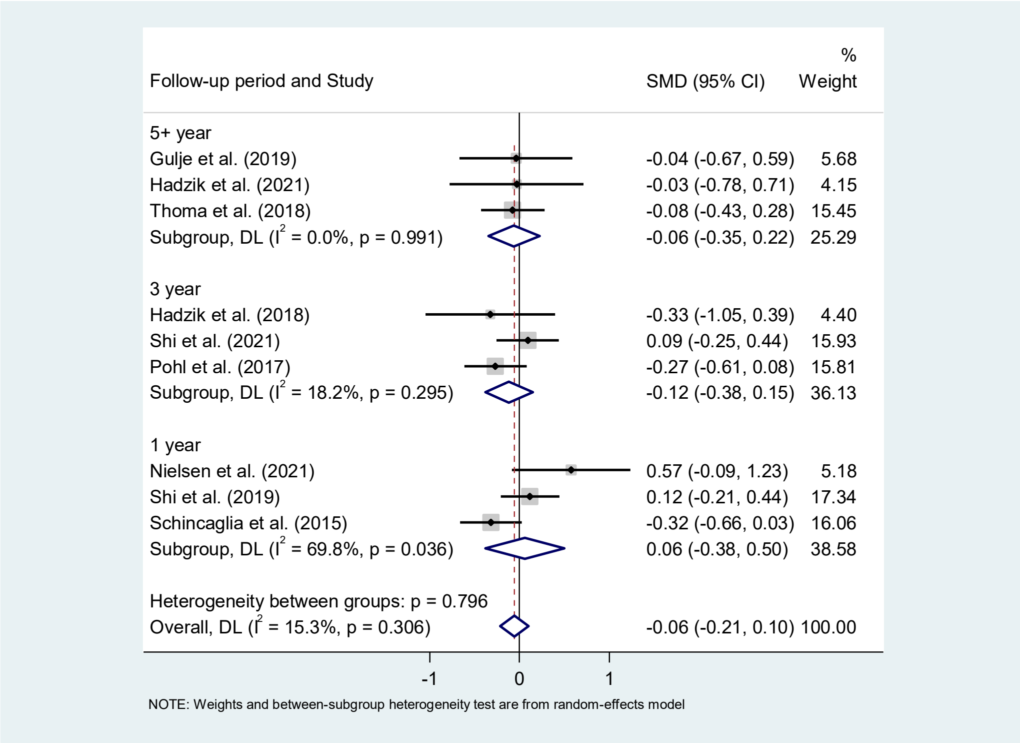

Figure S15 presents the combined random-effect of the mean difference in MBL (mm) between short and long groups, according to implant location-Mx post, stratified by follow-up period. In total, 9 studies were included in the present meta-analysis. The overall combined effect shows that the total mean difference in MBL (mm) between short and long groups was associated with a reduced mean difference in MBL in short implants (SMD: -0,06 95% CI (-0.21 to 0.10, z= -0.733, p-value=0.796> 0.05), however non-significant. No significant heterogeneity is present in the overall effect: I^2 = 15.3%, p-value= 0.306> 0.05).

Following, the combined effect for the follow-up period of 1 year, showed that the total mean difference in MBL (mm) between short and long groups was associated with an increased mean difference in MBL in short implants, however non-significant (SMD: 0,06 and corresponding 95% CI (-0.38 to 0.50, z= 0.264, p-value> 0.05). Regarding the follow-up period of 3 and 5+ years, the pooled estimate showed that the total mean difference in MBL (mm) between short and long groups was associated with a reduced mean difference in MBL in short implants, but is not statistically significant (SMDs: -0.12 and 0,06 with corresponding 95% CI (-0.38 to 0.15, z= -0.866, and -0.35 to 0.22, z= -0.431; p-values> 0.05). No significant heterogeneity is present in the follow-up period of 3 and 5+years: I^2 = 0% to 18.2%; p-values> 0.05), in contrast to the

studies with 1 year follow-up in which significant heterogeneity is present: $I^2 = 69.8\%$ and p-value= 0.036< 0.05.

Figure S16 Forest plot applying random-effect meta-analysis, assessing the risk difference of technical complications between short and long groups (data available in N= 9 studies).

Figure S16 presents the combined random-effect result, assessing the risk difference of technical complications in implants and in patients, between short and long groups. Therefore, regarding implants, the combined random-effect shows that the risk difference between short and long groups is associated with increased technical complications in the long group (risk difference %: 1% and 95% CI (-4% to 6%, z= 0.366 and p>0.05), however non-significant. On the other hand, when taking into account patients the combined random-effect shows that the risk difference between short and long groups is associated with reduced technical complications in the long group (risk difference %: 5% and 95% CI (-21% to 10%, z= -0.676 and p>0.05), but non-significant. In both cases, significant heterogeneity is not present: $I^2 >$ 34% and p-values> 0.05.

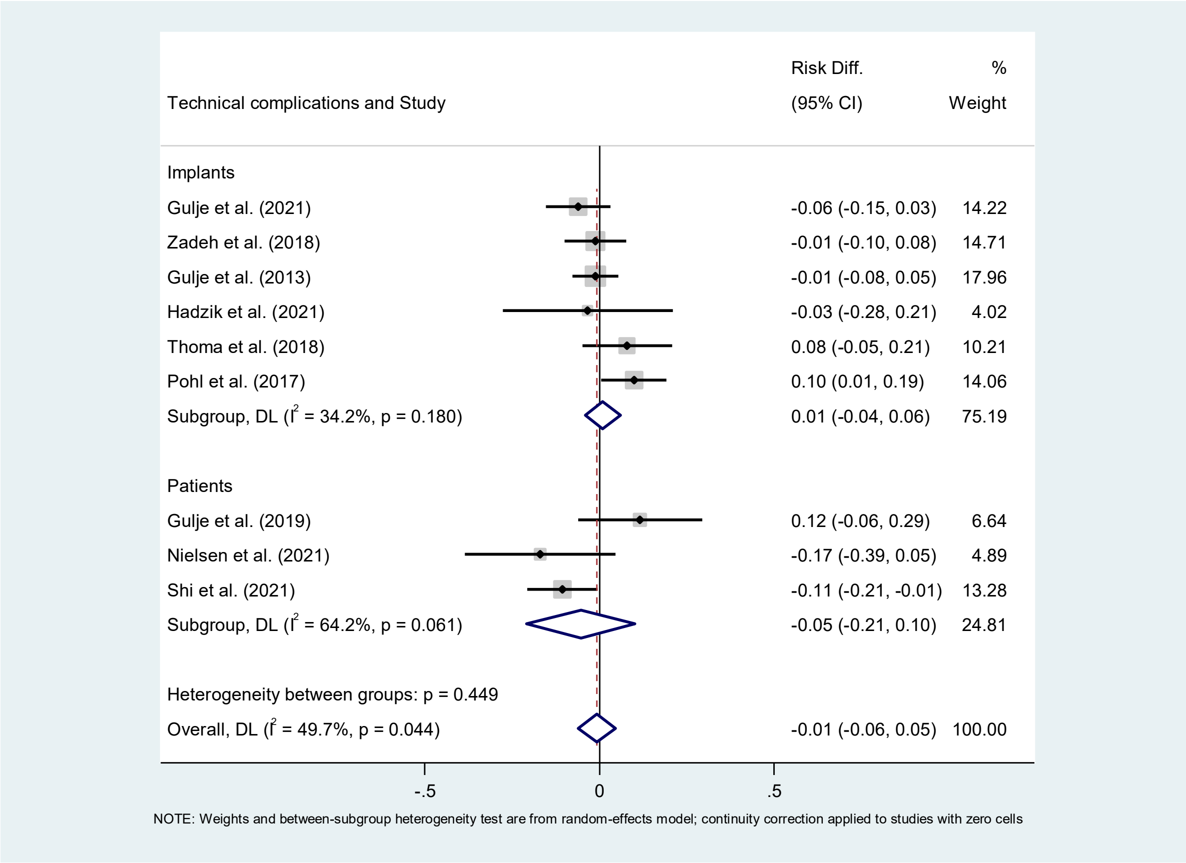

Figures S17 and S18 present the results from the random-effect meta-analysis assessing the mean value of MBL (mm), separately in the short and long group, by follow-up year and in total. Figures A3 and A4 show the results from the random-effect meta-analysis assessing the survival rates, also separately for short and long group, by follow-up period and in total.

Figure S17. Forest plot applying random-effect meta-analysis, assessing the mean value in MBL (mm) in short group and by follow-up period.

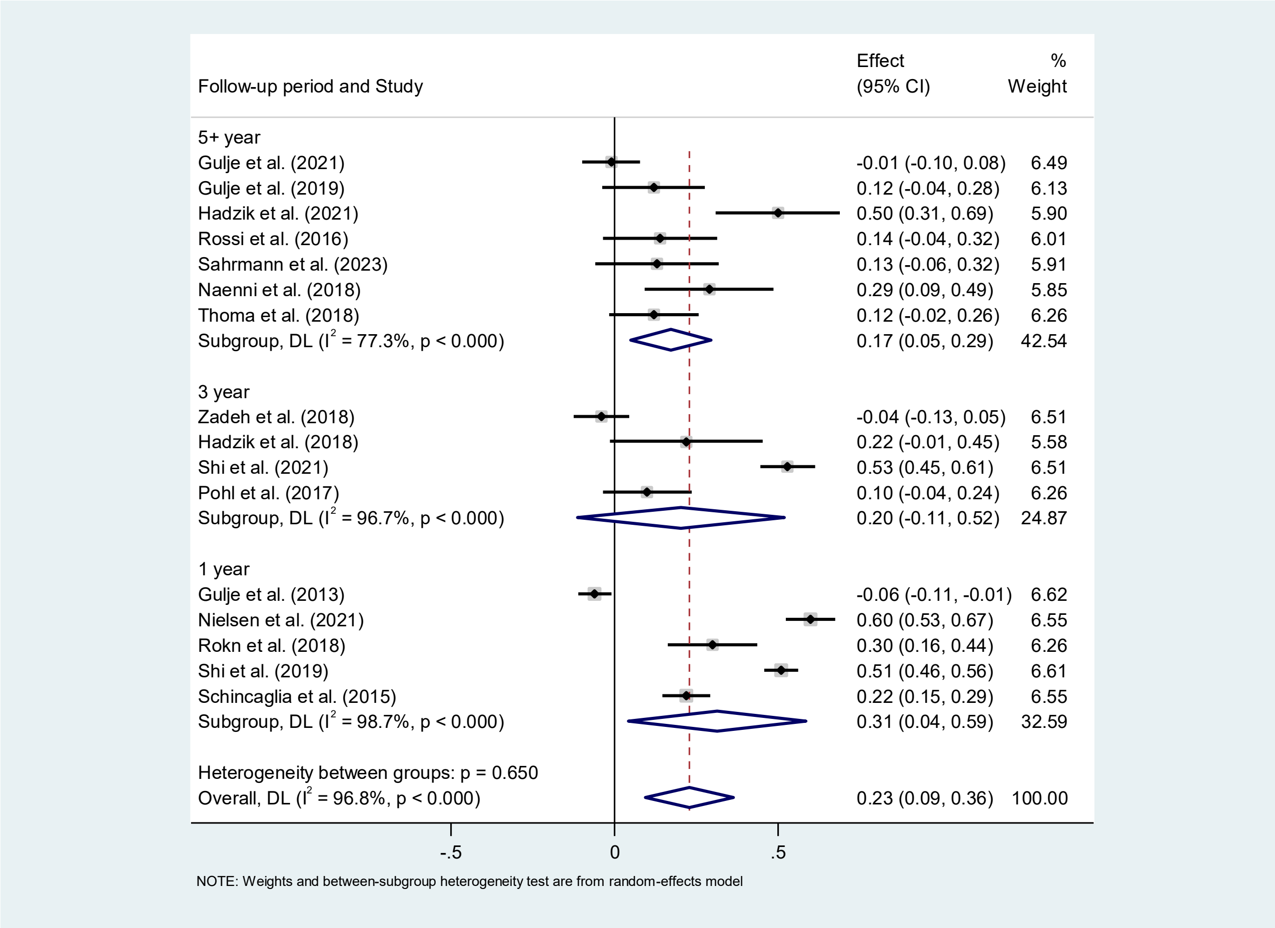

Figure S18. Forest plot applying random-effect meta-analysis, assessing the mean value in MBL (mm) in long group and by follow-up period.

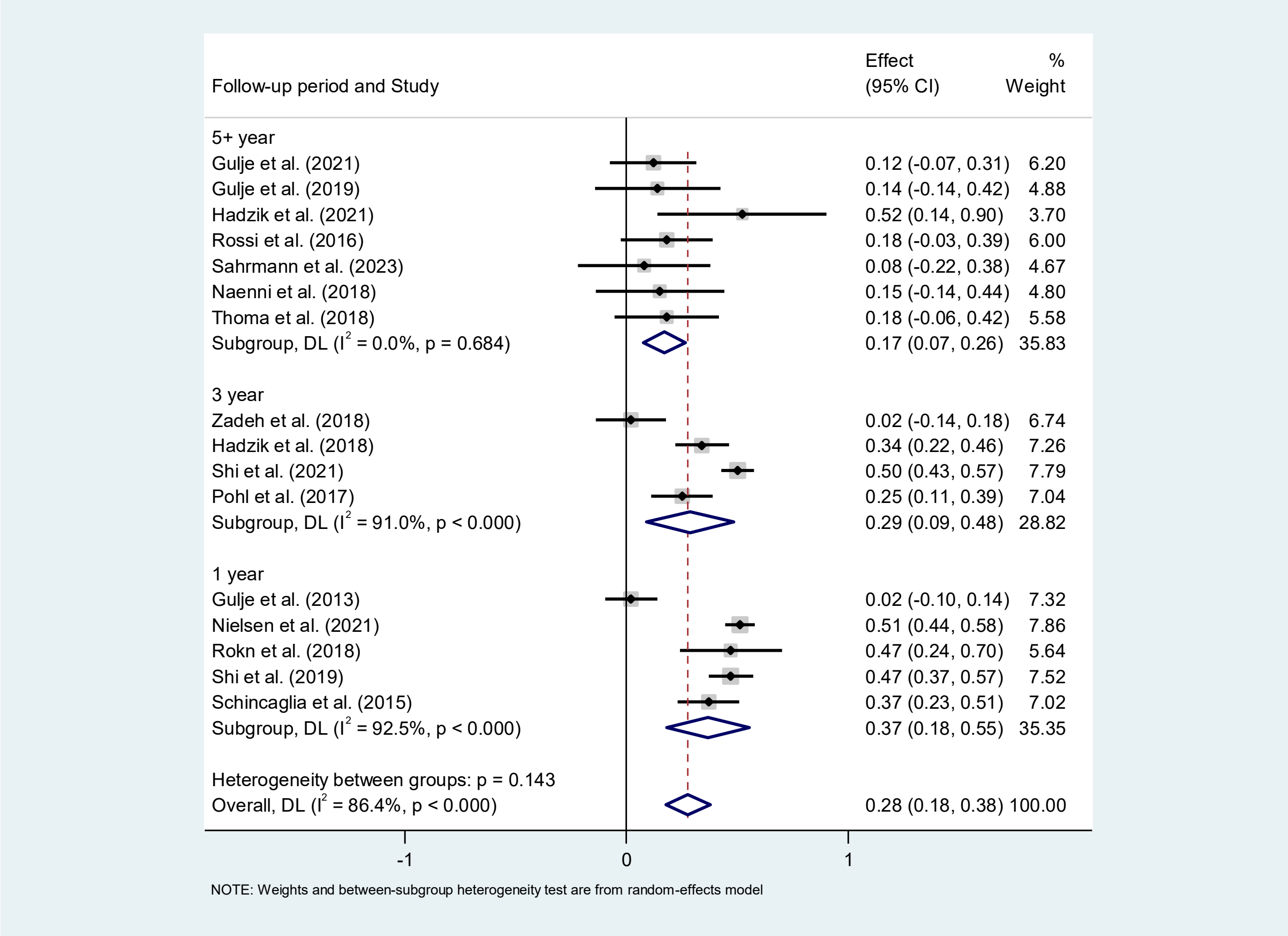

Figure S19. Forest plot applying random-effect meta-analysis, assessing the survival rate in short group and by follow-up period.

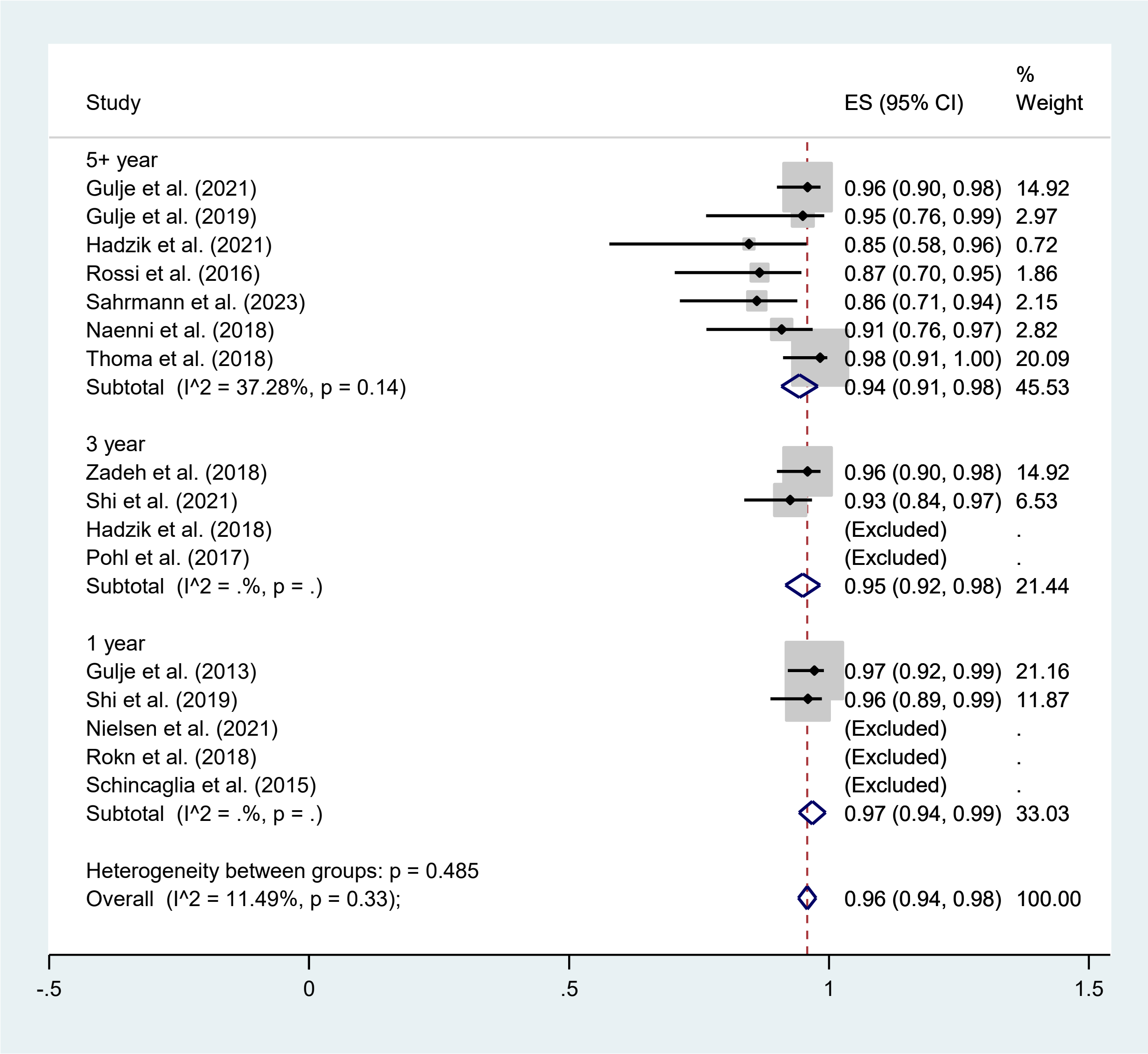

Figure S20. Forest plot applying random-effect meta-analysis, assessing the survival rate in long group and by follow-up period.

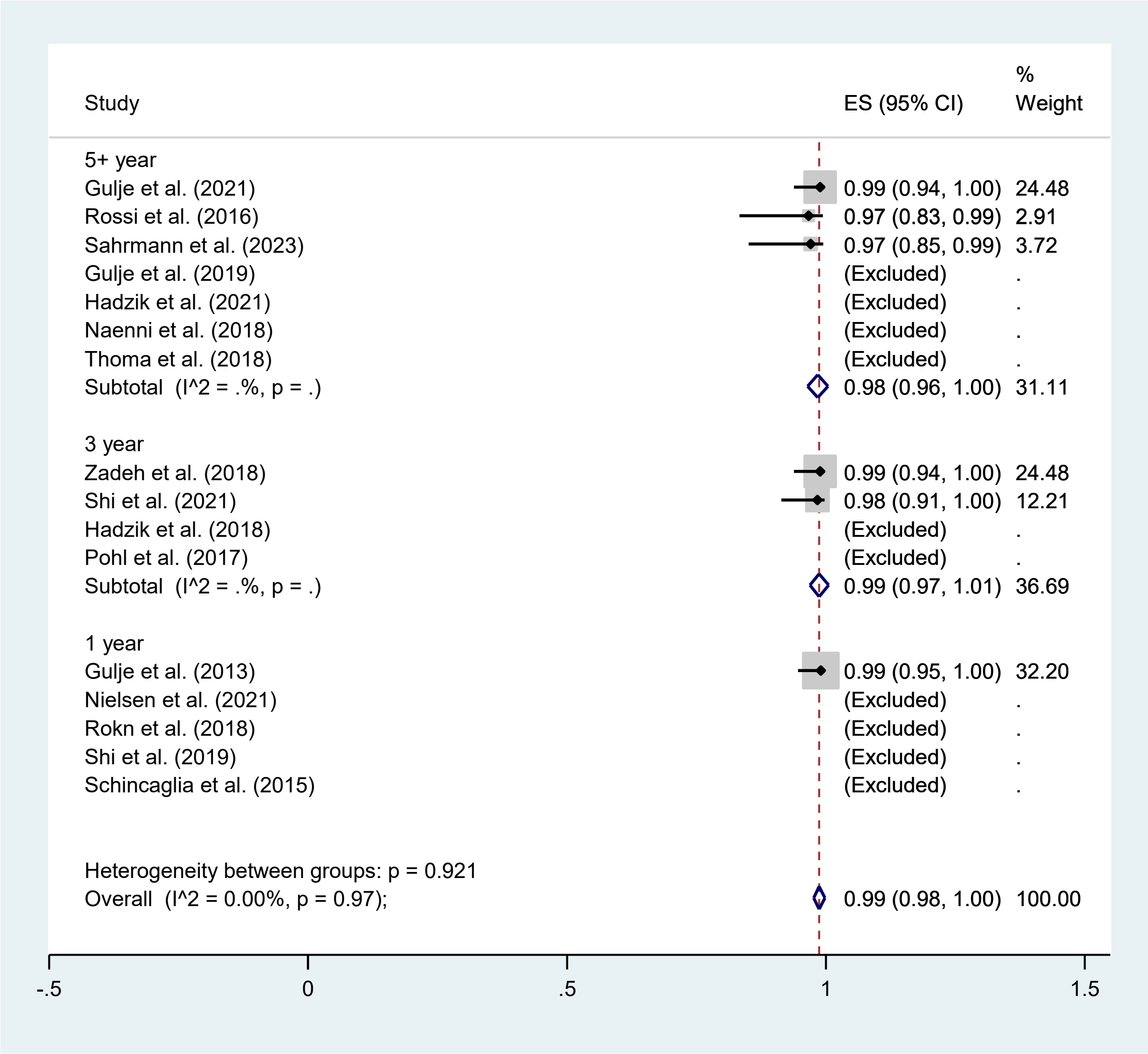

Table S1. Randomized clinical trials included in the present meta-analysis, regarding survival rate (%) difference between short and long group.

	Short		Long	
**Study**	**N**	**Survival rate (%)**	**N**	**Survival rate (%)**
Gulje et al. (2021)	98	96	88	99
Zadeh et al. (2018)	98	96	88	99
Gulje et al. (2013)	107	97	101	99
Gulje et al. (2019)	20	95	19	100
Hadzik et al. (2021)	13	87	15	100
Hadzik et al. (2018)	15	100	15	100
Nielsen et al. (2021)	20	100	17	100
Rokn et al. (2018)	24	100	21	100
Rossi et al. (2016)	30	87	30	97
Sahrmann et al. (2023)	36	86	34	97
Naenni et al. (2018)	33	91	45	100
Shi et al. (2021)	67	92	62	99
Shi et al. (2019)	74	96	70	100
Thoma et al. (2018)	60	99	64	100
Pohl et al. (2017)	61	100	68	100
Schincaglia et al. (2015)	63	100	69	100

Table S1 presents the number of patients in each group (short and long) and the survival rates of the 16 randomized clinical trial studies included in the present meta-analysis.

Table S2 Results from fixed-effect meta-analysis, regarding survival rate (%) value difference between short and long groups.

**Study**	**ES**	**[95% Conf. Interval]**	**% Weight**
Gulje et al. (2021)	0.03	[0.01 , 0.08]	10.55
Zadeh et al. (2018)	0.03	[0.01 , 0.08]	10.55
Gulje et al. (2012)	0.02	[0.01, 0.07]	15.67
Gulje et al. (2019)	0.05	[0.02 , 0.11]	6.47
Hadzik et al. (2021)	0.13	[0.08 , 0.21]	2.72
Rossi et al. (2015)	0.10	[0.06 , 0.17]	3.41
Sahrmann et al. (202	0.11	[0.06 , 0.19]	3.14

Naenni et al. (2018)	0.09	[0.05 , 0.16]	3.75
Shi et al. (2021)	0.07	[0.03 , 0.14]	4.72
Shi et al. (2019)	0.04	[0.02 , 0.10]	8.00
Thoma et al. (2018)	0.01	[0.00 , 0.05]	31.02
Hadzik et al. (2018)	(Excluded)		
Nielsen et al. (2021	(Excluded)		
Rokn et al. (2018)	(Excluded)		
Pohl et al. (2017)	(Excluded)		
Schincaglia et al. (	(Excluded)		
**Fixed pooled ES**	**0.04**	**[0.03 , 0.05]**	**100.00**

Test of ES=0 : z= 6.51 p = 0.00

Table S3. Randomized clinical trials included in the present meta-analysis, regarding MBL (mm) mean value difference between short and long group.

	**Short**			**Long**		
**Study**	**N**	**Mean**	**SD**	**N**	**Mean**	**SD**
Gulje et al. (2021)	98	-0.01	0.45	88	0.12	0.93
Zadeh et al. (2018)	98	-0.04	0.43	88	0.02	0.76
Gulje et al. (2013)	107	-0.06	0.27	101	0.02	0.6
Gulje et al. (2019)	20	0.12	0.36	19	0.14	0.63
Hadzik et al. (2021)	13	0.5	0.35	15	0.52	0.75
Hadzik et al. (2018)	15	0.22	0.46	15	0.34	0.24
Nielsen et al. (2021)	20	0.6	0.17	17	0.51	0.14
Rokn et al. (2018)	24	0.3	0.34	21	0.47	0.54
Rossi et al. (2016)	30	0.14	0.49	30	0.18	0.58
Sahrmann et al. (2023)	36	0.13	0.58	34	0.08	0.89
Naenni et al. (2018)	33	0.29	0.58	45	0.15	0.99
Shi et al. (2021)	67	0.53	0.35	62	0.5	0.3
Shi et al. (2019)	74	0.51	0.23	70	0.47	0.43
Thoma et al. (2018)	60	0.12	0.54	64	0.18	0.96
Pohl et al. (2017)	61	0.1	0.54	68	0.25	0.58
Schincaglia et al. (2015)	63	0.22	0.3	69	0.37	0.59

SD: Standard Deviation

Table S3 presents the number of patients in each group (short and long), the mean values of MBL (mm) and SD of the 16 randomized clinical trial studies included in the present meta-analysis.

Table S4. Results from fixed-effect meta-analysis, regarding MBL (mm) mean value difference between short and long groups.

**Study**	**SMD**	**[95% Conf. Interval]**	**% Weight**
Gulje et al. (2021)	-0.181	[-0.469,0.107]	11.46
Zadeh et al. (2018)	-0.099	[-0.387,0.189]	11.49
Gulje et al. (2013)	-0.174	[-0.446,0.099]	12.84
Gulje et al. (2019)	-0.039	[-0.667,0.589]	2.42
Hadzik et al. (2021)	-0.033	[-0.776,0.709]	1.73
Hadzik et al. (2018)	-0.327	[-1.048,0.394]	1.83
Nielsen et al. (2021	0.573	[-0.087,1.234]	2.19
Rokn et al. (2018)	-0.383	[-0.974,0.209]	2.73
Rossi et al. (2016)	-0.075	[-0.581,0.432]	3.72
Sahrmann et al. (2023)	0.067	[-0.402,0.536]	4.34
Naenni et al. (2018)	0.166	[-0.284,0.616]	4.71
Shi et al. (2021)	0.092	[-0.254,0.437]	7.98
Shi et al. (2019)	0.117	[-0.210,0.444]	8.91
Thoma et al. (2018)	-0.076	[-0.429,0.276]	7.68
Pohl et al. (2017)	-0.267	[-0.614,0.080]	7.91
Schincaglia et al. (2015)	-0.316	[-0.660,0.027]	8.07
**I-V pooled SMD**	**-0.087**	**[-0.184,0.011]**	**100**

Heterogeneity chi-squared = 12.97 (d.f. = 15) p = 0.604
I-squared (variation in SMD attributable to heterogeneity) = 0.0%

Test of SMD=0 : z= -1.74 p =0.082

details are provided regarding the output of all meta-analysis performed.

Table S4 presents the combined fixed-effect of all 16 RCTs. The combined effect shows that the total mean difference in MBL (mm) between short and long groups was associated with a reduced mean difference in MBL in short implants (standardized mean difference – SMD: -0,09 and corresponding 95% Confidence Interval - CI (-0.18 to 0.01, z= 1.74 and p= 0.082> 0.05).
